# Modular pathway engineering of key precursor supply pathways for lacto-*N*-neotetraose production in *Bacillus subtilis*

**DOI:** 10.1186/s13068-019-1551-3

**Published:** 2019-09-09

**Authors:** Xiaomin Dong, Nan Li, Zhenmin Liu, Xueqin Lv, Jianghua Li, Guocheng Du, Miao Wang, Long Liu

**Affiliations:** 1 Key Laboratory of Food Science and Technology, Ministry of Education, Jiangnan University, Wuxi, 214122 China; 2 State Key Laboratory of Dairy Biotechnology, Shanghai Engineering Research Center of Dairy Biotechnology, Dairy Research Institute, Bright Dairy & Food Co, Ltd, Shanghai, 200436 China; 3 Key Laboratory of Carbohydrate Chemistry and Biotechnology, Ministry of Education, Jiangnan University, Wuxi, 214122 China; 4 Key Laboratory of Industrial Biotechnology, Ministry of Education, Jiangnan University, Wuxi, 214122 China

**Keywords:** Lacto-*N*-neotetraose, *Bacillus subtilis*, Modular pathway engineering, Human milk oligosaccharides

## Abstract

**Background:**

Lacto-*N*-neotetraose (LNnT) is one of the important ingredients of human milk oligosaccharides, which can enhance immunity, regulate intestinal bacteria and promote cell maturation.

**Results:**

In this study, the synthetic pathway of LNnT was constructed by co-expressing the lactose permease (LacY) β-1,3-*N*-acetylglucosaminyltransferase (LgtA) and β-1,4-galactostltransferase (LgtB) in *Bacillus subtilis*, resulting in an LNnT titer of 0.61 g/L. Then, by fine-tuning the expression level of LgtB, the growth inhibition was reduced and the LNnT titer was increased to 1.31 g/L. In addition, by modular pathway engineering, the positive-acting enzymes of the UDP-GlcNAc and UDP-Gal pathways were strengthened to balance the two key precursors supply, and the LNnT titer was improved to 1.95 g/L. Finally, the LNnT titer reached 4.52 g/L in a 3-L bioreactor with an optimal glucose and lactose feeding strategy.

**Conclusions:**

In general, this study showed that the LNnT biosynthesis could be significantly increased by optimizing enzymes expression levels and modular pathway engineering for balancing the precursors supply in *B. subtilis*.

## Background

As one of the key components of human milk oligosaccharides (HMOs), Lacto-*N*-neotetraose (LNnT) is very beneficial for breast-feeding infant [1], which has the biological functions of enhancing immunity [2], regulating intestinal flora [3–7], and promoting cell maturation [8]. At present, as a potential nutraceutical, LNnT has been approved by the Food and Drug Administration of USA and the European Union to be added to infants and young children foods. LNnT is a linear tetrasaccharide (Galβ1-4GlcNAcβ1-3Galβ1-4Glc) consisting of d-galactose, *N*-acetylglucosamine, d-galactose, and d-glucose. Moreover, LNnT is also a core structure of other complex components of breast milk oligosaccharides [9]. These have promoted the growth of the market demand for LNnT. Currently, LNnT can be produced in large scale by chemical synthesis, but the multi-step reactions and expensive raw materials have led to its high price on dairy applications [10]. Compared with chemical synthesis, biosynthesis utilizes only cheap carbon sources and intracellular renewable donors. Therefore, biosynthetic LNnT has attracted the attention of many researchers.

In the previous study, the biosynthetic pathway of LNnT was constructed in *Escherichia coli* JM109 (the *lacZ* gene was knocked out) by overexpressing of β-1,3-*N*-acetylglucosaminyltransferase (LgtA) and β-1,4-galactostltransferase (LgtB) from *Nesseria meningitides* and lactose permease (LacY) [11]. However, the overexpression of LgtA and LgtB severely inhibits the normal growth of cells, which limited the efficient synthesis of LNnT. Moreover, because of the endotoxin content of *E. coli*, it is not the ideal host for producing the additive for infant formula. Unlike *E. coli*, *Bacillus subtilis*, a generally regarded as safe (GRAS) host, is highly attractive because of its many excellent characteristics, such as highly efficient protein synthesis system, genetically tractable, various metabolic engineering tools, and easy industrial scale production [12–17]. Thus, *B. subtilis* has been considered as chassis cell for microbial production of value-added products.

**Fig. 1:**
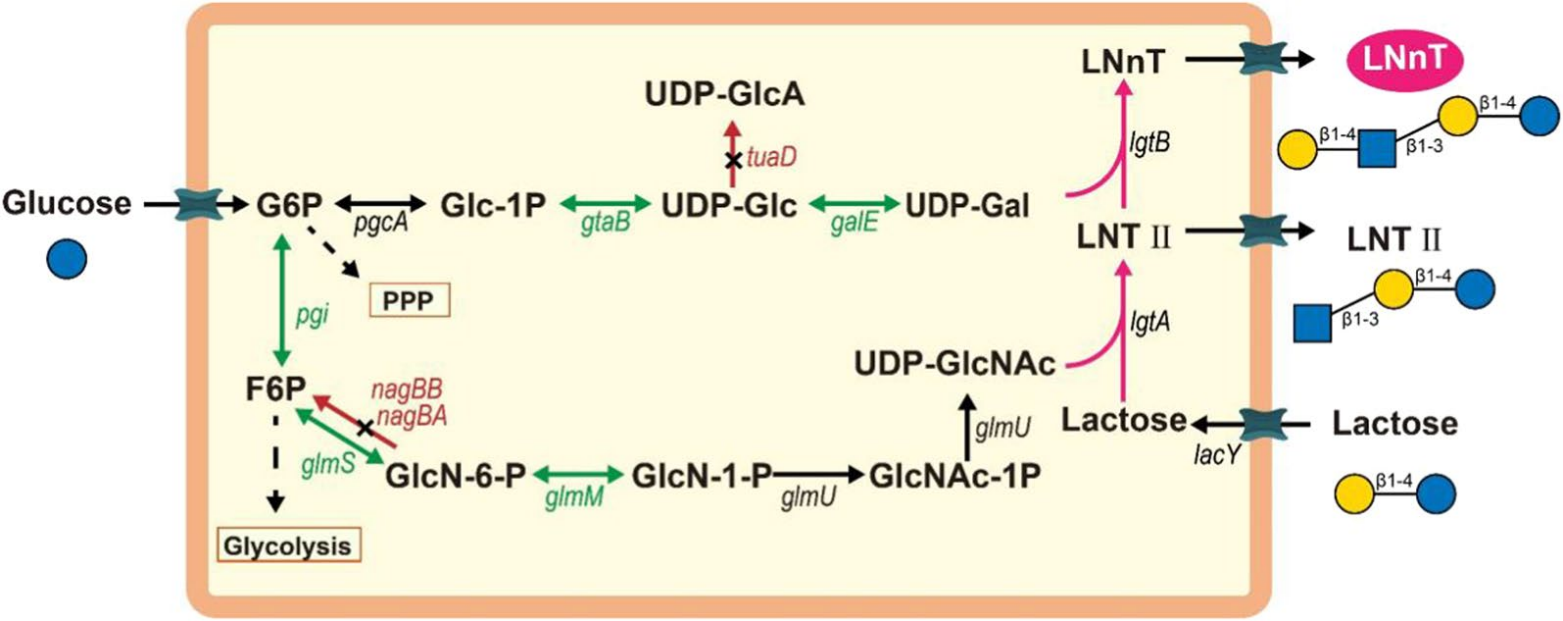
LNnT biosynthesis model in *B. subtilis*. black-colored arrows with dash line indicate several steps in the pathway. Green-colored arrows indicate the enhancing expressing of genes. Red-colored arrows indicate the deletion of genes. Pink-colored arrows indicate the construction of LNnT synthesis pathway. PEP, phosphoenolpyruvic acid; Pyr, pyruvate; G6P, glucose-6-phosphate; F6P, fructose-6-phosphate; GlcN-6-P, glucosamine-6-phosphate; GlcN-1-P, glucosamine-1-phosphate; GlcNAc-1P, *N*-acetylglucosamine-1-phosphate; UDP-GlcNAc, UDP-*N*-acetyglucosamine; Glc-1P, glucose-1-phosphate; UDP-Glc, UDP-glucose; UDP-GlcA, UDP-glucuronate; UDP-Gal, UDP-galactose; LNTII, Lacto-*N*-triose II; LNnT, Lacto-*N*-neotetraose. *pgi*, encoding glucose-6-phosphate isomerase; *glmS*, encoding glucosamine-6-phosphate synthase; *glmM*, encoding phosphoglucosamine mutase; *glmU*, encoding *N*-acetylglucosamine-1-phosphate uridyltransferase/glucosamine-1-phosphate acetyltransferase; *nagBA*, encoding glucosamine-6-phosphate deaminase 1; *nagBB*, encoding glucosamine-6-phosphate deaminase 2; *pgcA*, encoding phosphoglucomutase; *gtaB*, encoding UTP-glucose-1-phosphate uridylyltransferase; *galE*, encoding UDP-glucose 4-epimerase; *tuaD*, UDP-glucose 6-dehydrogenase; *lacY*, lactose permease; *lgtA*, β-1,3-*N*-acetylglucosaminyltransferase; *lgtB*, β-1,4-galactostltransferase;  : Glucose;  : *N*-acetylglucosamine;  : Galactose

In this study, *B. subtilis* 168 was selected as the host and the de novo LNnT synthesis pathway was constructed by co-expression of LacY, LgtA, and LgtB. As shown in Fig. 1, UDP-*N*-acetyglucosamine (UDP-GlcNAc) and lactose are catalyzed by LgtA to produce Lacto-*N*-triose II (LNTII), and then, the LNTII and UDP-galactose (UDP-Gal) are catalyzed into LNnT by LgtB. Therefore, two key factors might affect the efficient synthesis of LNnT: 1) the cell growth inhibited by co-overexpressing of heterologous proteins and 2) the supply of key precursors of UDP-GlcNAc and UDP-Gal. In view of the above problems, we first identified the growth inhibition caused by overexpression of heterologous enzymes, and optimized the expression level of LgtB to alleviate cell growth inhibition, resulting in the increase of LNnT titer to 1.31 g/L in shake flasks. Next, the supply of two precursors was further enhanced and balanced by modular pathway engineering, which boosted the LNnT titer from 1.31 g/L to 1.95. Finally, by optimizing the glucose and lactose feeding strategy in a 3-L bioreactor, the LNnT titer increased to 4.52 g/L, which was 2.3-fold over that in shake flask. The three strategies used in this study for reducing growth inhibition via optimizing protein expression levels, balancing the precursors supply via modular pathway engineering, and optimization of carbon sources feeding strategy in 3-L bioreactor provide a good start for the effective production of LNnT in *B. subtilis*. Moreover, LNnT was secreted into the fermentation broth, which was convenient for isolation and suitable for industrial production.

## Materials and methods

### Microorganisms and cultivation conditions

*Bacillus subtilis* 168 strain was used as the initial host. The recombinant *B. subtilis* used in this study are listed in Table 1. *E. coli* JM109 was used as host for gene cloning. The p7Z6, p7S6, p7C6, p7Z6P43, p7S6P43, p7C6P43, pDG-*Cre*, and pP43NMK were stored in our laboratory.

**Table 1:** Strains and plasmids used in this study

Names	Characteristics	References
Strains		
BS168*comK*	*B. subtilis* 168 derivate, P_*xylA*_-*comK*	This work
BY01	BS168*comK* derivate, *ΔamyE*::P_*43*_-*lacY*	This work
BY02	BY01 derivate, P_*43*_-*lgtA*, P_*43*_-*lgtB*	This work
BY03	BY01 derivate/pP43NMK-*lgtA*-*lgtB*	This work
BY04	BY01 derivate, P_*43*_-*lgtB*/pP43NMK-*lgtA*	This work
BY05	BY04 derivate, P_*43*_-lgtB	This work
BY06	BY05 derivate, P_*43*_-lgtB	This work
BY07	BY06 derivate, P_*43*_-lgtB	This work
BY08	BY06 derivate, P_*43*_-*pgi*	This work
BY09	BY06 derivate, P_*43*_-*glmS*	This work
BY10	BY06 derivate, P_*43*_-*glmM*	This work
BY11	BY06 derivate, P_*43*_-*glmU*	This work
BY12	BY06 derivate, *ΔnagBAΔnagBB*::*lox72*	This work
BY13	BY04 derivate, P_*43*_-*pgi*	This work
BY14	BY04 derivate, P_*43*_-*pgcA*	This work
BY15	BY04 derivate, P_*43*_-*gtaB*	This work
BY16	BY04 derivate, *ΔtuaD*	This work
BY17	BY04 derivate, P_*43*_-*galE*	This work
BY18	BY06 derivate, P_*43*_-*pgi*, P_*43*_-*gtaB*	This work
BY19	BY18 derivate, P_*43*_-*glmS*	This work
BY20	BY19 derivate, P_*43*_-*glmM*	This work
BY21	BY20 derivate, *ΔnagBAΔnagBB*::*lox72*	This work
BY22	BY18 derivate, P_*43*_-*galE*	This work
BY23	BY19 derivate, P_*43*_-*galE*	This work
BY24	BY20 derivate, P_*43*_-*galE*	This work
BY25	BY21 derivate, P_*43*_-*galE*	This work
BY26	BY22 derivate, *ΔtuaD*::*lox72*	This work
BY27	BY23 derivate, *ΔtuaD*::*lox72*	This work
BY28	BY24 derivate, *ΔtuaD*::*lox72*	This work
BY29	BY25 derivate, *ΔtuaD*::*lox72*	This work
BY30	BY26 derivate, P_*43*_-*pgcA*	This work
BY31	BY27 derivate, P_*43*_-*pgcA*	This work
BY32	BY28 derivate, P_*43*_-*pgcA*	This work
BY33	BY29 derivate, P_*43*_-*pgcA*	This work
Plasmids		
pDG-*Cre*	Km^r^, Amp^r^, temperature sensitive in *B. subtilis*	[15]
p7Z6	pMD18-T containing *lox71*-*zeo*-*lox66* cassette	[15]
p7S6 p7C6	pMD18-T containing *lox71*-*spc*-*lox66* cassette pMD18-T containing *lox71*-*cm*-*lox66* cassette	[15] [15]
p7Z6P43	p7Z6 containing P_*43*_ promoter	[22]
p7C6P43	p7C6 containing P_*43*_ promoter	[22]
p7S6P43	p7S6 containing P_*43*_ promoter	[22]
pP43-*lgtA*-*lgtB*	pP43NMK derivate with *lgtA* and *lgtB* cloned	This work
pP43-*lgtA*	pP43NMK derivate with *lgtA* cloned	This work

Luria–Bertaini (LB) medium was used for all *B. subtilis* strains and *E. coli* JM109 cultivation. The shake-flask fermentation medium contained (g/L): yeast extract 12, peptone 6, (NH_4_)_2_SO_4_ 6, K_2_HPO_4_·4H_2_O 12, KH_2_PO_4_ 2.5, MgSO_4_·7H_2_O 3, Urea 1.5, and glucose 60.

Shake-flask culture for the production of LNnT: 5 mL of seed solution cultured for 12 h in LB was added to baffled 500 mL shake flask with 45 mL of fermentation medium. Fermentation conditions are 37 °C, 220 rpm. 1 mL samples were taken during fermentation for determining OD_600_.

### Plasmid construction

The plasmids are listed in Table 1, and all primers are listed in Additional file 1: Table S1. The *lgtA* (Genbank ID: 904226) and *lgtB* (Genbank ID: 904227) from *Neisseria meningitidis* MC58 were codon optimized and synthetized by Nanjing Genscript Biotech Company (Nanjing, china). DNA sequences of the codon-optimized genes are listed in Additional file 1: Tables S2, S3. To construct the pP43NMK-*lgtA*-*lgtB* plasmid, the *lgtA* gene and *lgtB* gene were cloned into pP43NMK plasmid. To construct the pP43NMK-*lgtA* plasmid, the *lgtA* gene was cloned into pP43NMK plasmid using the Gibson Assembly Kit (New England Biolabs, NEB).

### LNnT biosynthesis pathway construction

All of the primers used are listed in Additional file 1: Table S1. The *Cre*/*lox* system was selected for integration into the genome and gene knockout [18]. The DNA recombination fragment was obtained by overlap extension-PCR, and transformed into *B. subtilis* by the comK method [19]. The antibiotics including ampicillin (30 μg/mL), kanamycin (30 μg/mL), zeocin (30 μg/mL), chloramphenicol (5 μg/mL), and spectinomycin (50 μg/mL) were used for selections.

The original promoter of the *comk* gene was replaced with a xylose-inducible promoter P_*xylA*_ from the strain *B. subtilis* 168, yielding the strain BS168*comk*. *lacY gene* from *E. coli* K12 was integrated into the genome of BS168*comk*, yielding that the strain BY01. *lgtA* and *lgtB* genes were integrated into the genome of BY01, producing the strain BY02. Plasmid pP43NMK-*lgtA*-*lgtB* was transformed into BY01, yielding the strain BY03. One, two, three, and four copies of *lgtB* were integrated into the genome and plasmid pP43NMK-*lgtA* was transformed into BY01, yielding the strain BY04, BY05, BY06, and BY07, respectively. The genomic loci of *lgtA* and *lgtB* genes integration expression are listed in Additional file 1: Tables S4–S8.

### Knockout of *tuaD*, *nagBA*, *nagBB*, overexpression of *pgcA*, *gtaB*, *galE*, *pgi*, *glmS*, *glmM*, *glmU*

To accelerate the conversion between intermediate metabolites in the UDP-Gal precursor pathway, *pgcA* encoding phosphoglucomutase, *gtaB* encoding UTP-glucose-1-phosphate uridylyltransferase, and *galE* encoding UDP-glucose 4-epimerase were overexpressed under the control of P_*43*_ promoter. To block the conversion of UDP-Glc to UDP-glucuronate, *tuaD* encoding UDP-glucose 6-dehydrogenase was deleted. To enhance the conversion between intermediate metabolites in the UDP-GlcNAc precursor pathway, *pgi* encoding glucose-6-phosphate isomerase*, glmS* encoding glucosamine-6-phosphate synthase, *glmM* encoding phosphoglucosamine mutase, and *glmU* encoding *N*-acetylglucosamine-1-phosphate uridyltransferase/glucosamine-1-phosphate acetyltransferase were overexpressed under the control of P_*43*_ promoter. To block the conversion of GlcN-6-P to F6P, *nagBA* encoding glucosamine-6-phosphate deaminase 1 and *nagBB* encoding glucosamine-6-phosphate deaminase 2 were simultaneously knocked out. The genomic loci of *pgcA*, *gtaB*, *galE*, *pgi*, *glmM,* and *glmU* genes integration expression are listed in Additional file 1: Tables S9–S14. The constructed strains BY08–BY17 and BY18–BY33 are listed in Table 1.

### Microbial production of LNnT in 3-L bioreactor

The production of LNnT by batch culture of BY28 strain was performed with the optimized fermentation medium containing: 30 g/L glucose, 1.8 g/L lactose, 20 g/L yeast extract, 20 g/L tryptone, 8 g/L urea, 12.5 g/L K_2_HPO_4_·3H_2_O, 2.5 g/L KH_2_PO_4_, and 10 mL/L trace metal solution. The trace metal solution contained 4 g/L FeSO_4_·7H_2_O, 4 g/L CaCl_2_, 1 g/L MnSO_4_·5H_2_O, 0.4 g/L CoCl_2_·6H_2_O, 0.2 g/L NaMoO4·2H_2_O, 0.2 g/L ZnSO_4_·7H_2_O, 0.1 g/L AlCl_3_·6H_2_O, 0.1 g/L CuCl_2_·H_2_O, and 0.05 g/L H_3_BO_4_. The feeding solution contained 500 g/L glucose and 30 g/L lactose. 180 mL of seed culture that was cultured in 1 L shake flasks for 12 h was added into a 3-L fermentor (BioFlo 115, New Brunswick Scientific Co., Edison, NJ, USA) containing initial 1.62 L of fermentation medium. Agitation was provided by 2 6-bladed disk turbines. The pH was automatically kept at 7.0 via the addition 14% NH_3_·H_2_O, the temperature was maintained at 37 °C, and the aeration rate and agitation speed were 1.0 vvm and 800 rpm, respectively.

In the intermittent feeding fermentation, whenever the residual glucose concentration was lower than 5 g/L, the feeding solution of glucose and lactose was added to the bioreactor to restore the glucose concentration to about 30 g/L. In the dual-flow continuous feeding strategy with control of glucose concentration, the glucose concentration was maintained at 10–20 g/L via adjusting the feeding rates based on the concentration of residual glucose in the bioreactor.

### Analytic methods

Samples were centrifuged at 14,000×*g* for 5 min. The supernatant was diluted 10 times with ddH_2_O after removing the protein by the sevage method, and the sevage solvent was replaced by chloroform/n-butanol (4:1 v/v) [20]. The concentrations of LNTII, LNnT, and lactose were measured by high-performance anion-exchange chromatography–pulsed amperometric detection (HPEAC–PAD) using a CarboPac PA10 (4 × 250 mm) column. The mobile phase was NaOH (36 mM) at a flow rate of 1.00 mL/min and 30 °C. The injection volume was 25 µL. OD_600_ was converted to dry cell weight (DCW) according to the following equation: 1OD_600_ = 0.35 g/L. The glucose concentration was measured using a glucose–glutamate analyzer (SBA-40C; Biology Institute of Shandong Academy of Sciences). All experiments were independently done at least three times and the statistical analyses were performed using the one-way ANOVA.

## Results and discussion

### Design and construction of LNnT biosynthesis pathway

We chose *B. subtilis* 168 as the initial strain. To improve the efficiency of transforming plasmids or integration cassettes, the original promoter of the *comk* gene was replaced with a xylose-inducible promoter, generating strain BS168*comK* [19]. Then, β-galactoside permease gene (*lacY*) from *E. coli* K12 was inserted into the genome and overexpressed using the strong constitutive promoter P_*43*_ for transporting the precursor lactose into *B. subtilis*. In the biosynthesis pathway in vivo, enzymes with effective catalytic capabilities are crucial for efficient production of LNnT. Considering that *lgtA* gene encoding β-1,3-*N*-acetylglucosaminyltransferase and *lgtB* gene encoding β-galactostltransferase from *Neisseria meningitidis* are known to have catalytic activity for LNnT synthesis [11, 21], we chose to introduce these two enzymes to construct LNnT synthesis pathway.

**Fig. 2:**
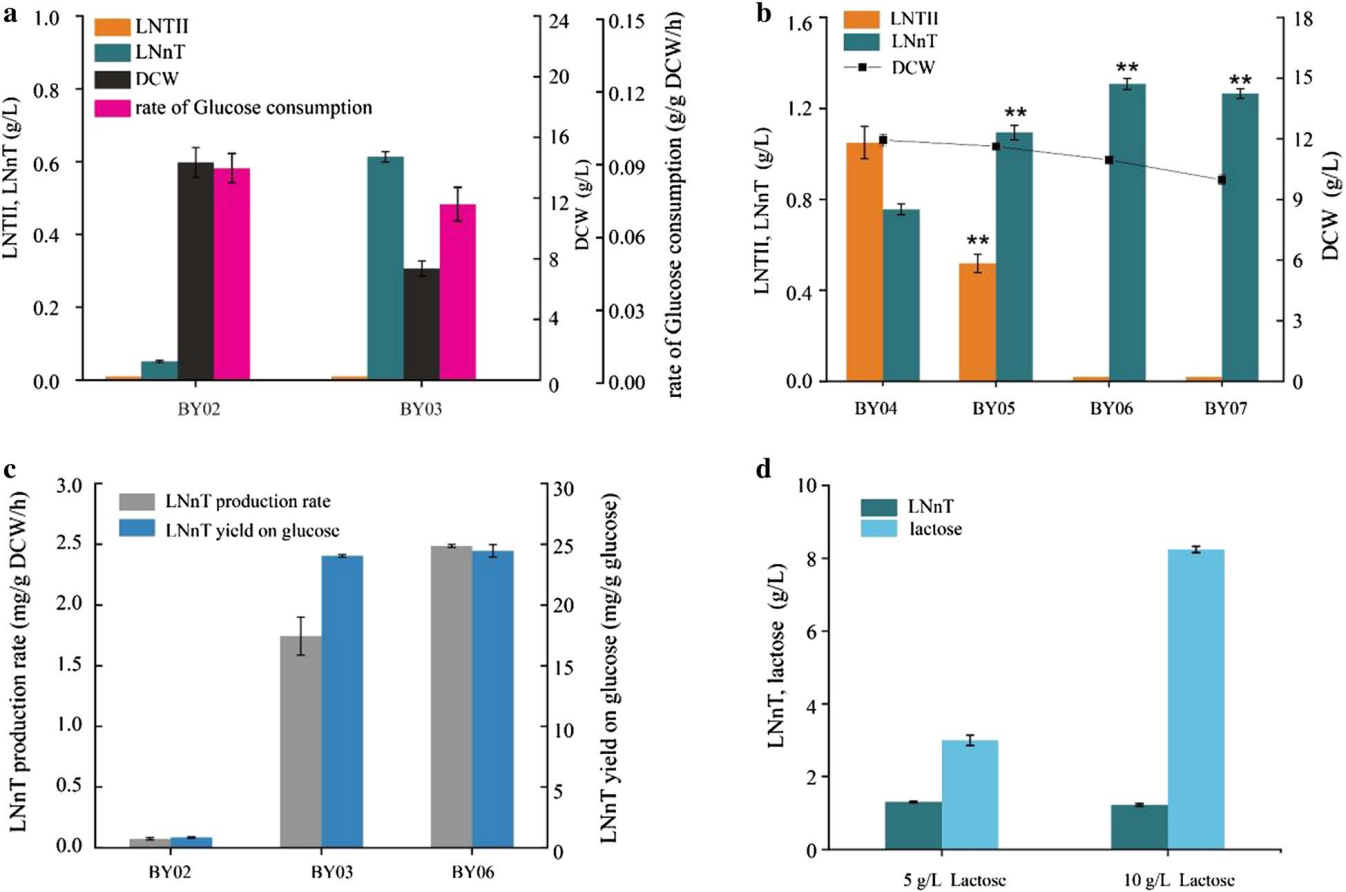
Effects of different expression levels of *lgtA* and *lgtB* genes. **a** LNTII, LNnT titer of BY02 and BY03, DCW and rate of glucose consumption in BY02 and BY03; **b** LNTII, LNnT titer of BY04–BY07, and DCW in BY04–BY07, the** mean p < 0.01; **c** LNnT production rate, and yield on glucose in BY02, BY03 and BY04; **d** LNnT titer and lactose concentration of BY06, when cultivated with 5 g/L lactose and 10 g/L lactose. Triplicate experiments were done, and error bars represent the standard deviation

Overexpression of key enzymes can improve catalytic activity; however, overexpression of protein may lead to growth inhibition and thus decrease synthesis efficiency [22]. Therefore, to reduce growth inhibition, optimizing the amount of heterologous proteins expression may be an ideal method. To identify the optimal expression of LgtA and LgtB, we tested the two key enzymes’ expression level via integrating different copy numbers or expressing on plasmid in engineered *B. subtilis*. The pathway for de novo LNnT biosynthesis was constructed by co-expressing LgtA and LgtB under the control of strong constitutive promoter P_*43*_ using integration expression system or pP43NMK plasmid, yielding BY02 and BY03, respectively. The titer of LNTII in BY02 and BY03 was both below detection level, which suggested that the high expression level of LgtA was still insufficient for LNnT synthesis (Fig. 2a). Therefore, the expression of LgtA in the pP43NMK plasmid system (high level) is more suitable for LNnT synthesis. Compared with controlling the expression of LgtB at the low level (strain BY02), the LNnT titer increased significantly by 12.2 fold to 0.61 g/L (strain BY03) when the expression of LgtB was increased to the highest level. However, compared with strain BY02 (DCW = 14.35 g/L, 0.087 g/g DCW/h), the maximum DCW value and glucose consumption rate of the BY03 strain (DCW = 7.35 g/L, 0.073 g/g DCW/h) were significantly reduced by 48.8% and 16.1%, respectively (Fig. 2a). We speculated that the co-overexpressed heterologous protein LgtB and LgtA arrested normal cell growth. To confirm our speculation, the expression level of LgtB was changed via integrating different copies (*n* = 1, 2, 3, 4) in the genome under P_*43*_ promoter, while the LgtA was expressed using the pP43NMK plasmid, yielding strains BY04, BY05, BY06, and BY07, respectively. Compared with strain BY04, BY05, and BY07, the titer of LNnT in strain BY06 was the highest and the most of LNT II in BY06 was converted to LNnT (Fig. 2b).

In this study, under the premise that LgtA must be overexpressed, the growth of cells was successfully restored by optimizing LgtB expression to an appropriate level, and the DCW raised from 7.35 g/L (strain BY03) to 11 g/L (strain BY06), and the titer of LNnT increased by 114.8% to 1.31 g/L. Furthermore, the LNnT yield on glucose (24.4 mg/g glucose) and the production rate (2.5 mg/g DCW/h) of strain BY06 were both higher than that of strain BY02 and BY03 (Fig. 2c). When the amount of lactose added to the fermentation medium increased from 5 g/L to 10 g/L, the yield of LNnT in the strain BY06 did not further increase, suggesting that the supply of lactose was sufficient when the amount of lactose was 5 g/L (Fig. 2d). However, the extracellular LNT II was below detection level in the strain BY06, so we speculated that intracellular precursors might limit efficient LNnT synthesis due to its insufficient supply.

### Design and construction of the UDP-GlcNAc and UDP-Gal supply module

In our previous study, the titer of hyaluronic acid was greatly increased by simultaneously increasing two precursors, UDP-glucuronate (UDP-GlcA) and UDP-GlcNAc via overexpressing the enzymes in the precursors’ synthesis pathways [23]. Therefore, next, we focus on how to improve and balance the supply of two precursors UDP-GlcNAc and UDP-Gal for LNnT synthesis. The precursors UDP-Gal and UDP-GlcNAc are derived from glucose 6-phosphate and fructose 6-phosphate of Embden–Meyerhof–Parnas pathway (EMP), respectively. Modular pathway engineering divides complex synthetic networks into modules, systematically strengthens and balances the various modules to optimize the entire metabolic network via assembling different strengths between modules [24, 25]. Therefore, modular pathway engineering can be used as an effective strategy to balance the UDP-GlcNAc and UDP-Gal supply for LNnT synthesis. In this work, we divided the LNnT biosynthetic pathway into the UDP-GlcNAc and UDP-Gal supply modules.

We attempted to identify positive-acting enzymes in the UDP-GlcNAc and UDP-Gal pathways by enhancing related gene expression through adding one copy in the genome under P_*43*_ promoter or blocking relevant gene expression. Specifically, the original promoter of *glmS* gene was replaced with P_*43*_ promoter. Specifically, the UDP-GlcNAc supply module contained *pgi* (encoding glucose-6-phosphate isomerase), *glmS* (encoding glucosamine-6-phosphate synthase), *glmM* (encoding phosphoglucosamine mutase), *glmU* (encoding *N*-acetylglucosamine-1-phosphate uridyltransferase/glucosamine-1-phosphate acetyltransferase), *nagBA* (encoding glucosamine-6-phosphate deaminase 1), and *nagBB* (encoding glucosamine-6-phosphate deaminase 2). The UDP-Gal synthesis pathway included *pgcA* (encoding phosphoglucomutase), *gtaB* (encoding UTP-glucose-1-phosphate uridylyltransferase), *galE* (encoding UDP-glucose 4-epimerase), and *tuaD* (UDP-glucose 6-dehydrogenase). We verified the effect of Pgi in the two modules, as Pgi is a bidirectional enzyme that can also convert fructose 6-phosphate to glucose 6-phosphate.

**Fig. 3:**
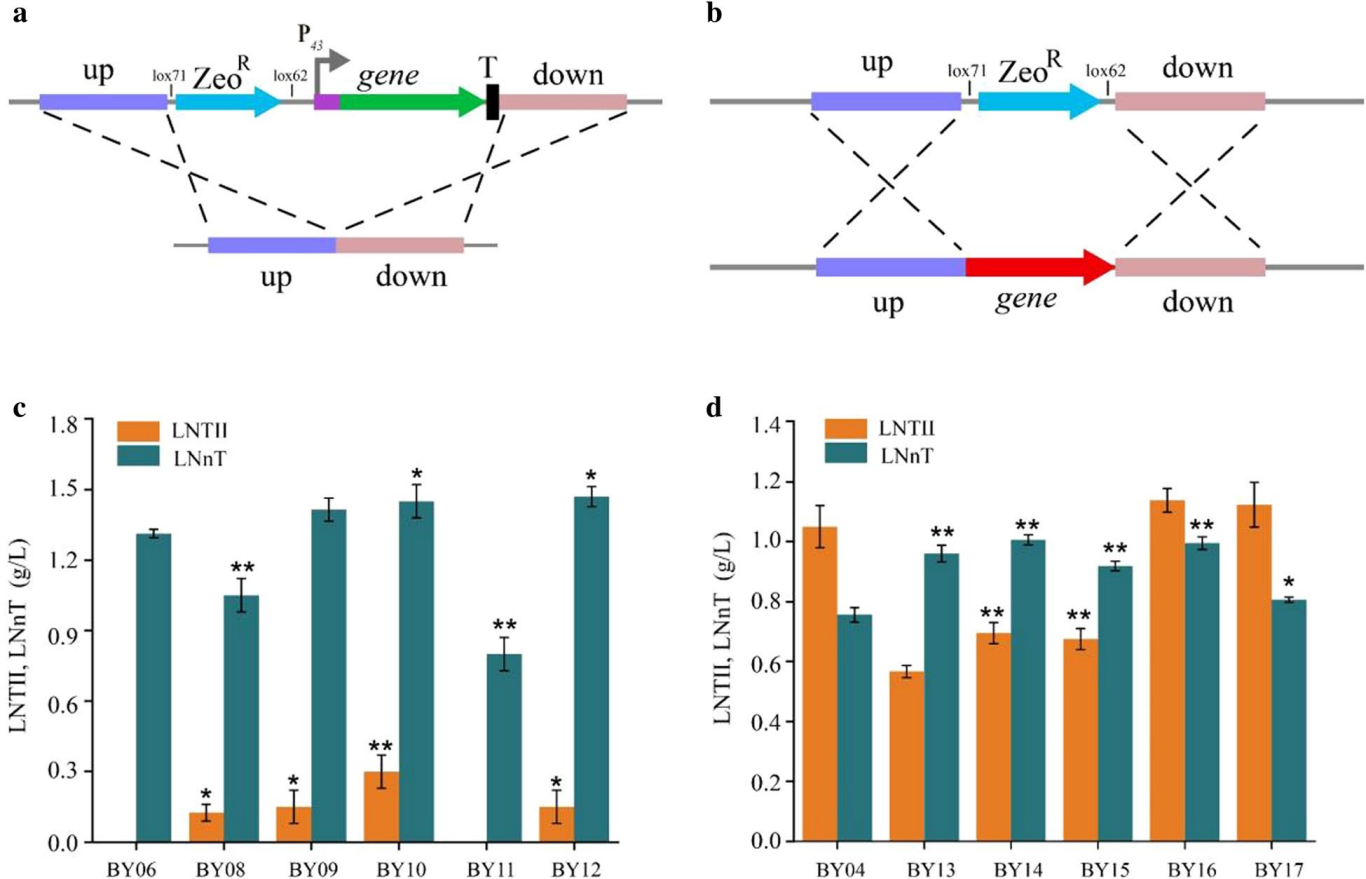
Identifying positive-acting enzymes in the UDP-GlcNAc and UDP-Gal pathways. **a** Schematic of the cassette used to overexpress gene in *B. subtilis*. **b** Schematic of the cassette used to knockout gene in *B. subtilis*. **c** LNTII, LNnT titer of BY08 ~ BY12 and the control host BY06. **d** LNTII, LNnT titer of BY13–BY17 and the control host BY04. Triplicate experiments were done, and error bars represent the standard deviation. * and** mean p < 0.05 and p < 0.01, respectively

To verify the positive-acting enzyme in the UDP-GlcNAc module, the UDP-GlcNAc precursor-deficient strain BY06 was engineered. As shown in Fig. 3c, the titer of LNnT increased from 1.31 g/L (BY06) to 1.42 g/L (BY09, overexpression of *glmS* gene), 1.45 g/L (BY10, overexpression of *glmM* gene), and 1.47 g/L (BY12, knocking out of *nagBA* and *nagBB* genes), respectively. The up-regulation of *glmS* and *glmM* and co-deletion of *nagBA* and *nagBB* in the branch pathways demonstrated that the conversion of F6P to GlcN-6-P (glucosamine-6-phosphate) and then to GlcN-1-P (glucosamine-1-phosphate) was the rate-limiting step for LNnT synthesis. The titer of LNTII increased to 0.13 g/L, while the titer of LNnT decreased to 1.05 g/L in BY08 (overexpression of *pgi* gene), indicating that up-regulation of *pgi* can lead to glucose flowing to UDP-GlcNAc synthetic pathway in BY06. However, the titer of LNnT was reduced by 38.9% in BY11 (overexpression of *glmU* gene), so we guessed that the overexpression of *glmU* was not suitable for the enhancement of the UDP-GlcNAc pathway in the LNnT-producing strain. The BY04 strain was used as the host for confirming rate-limiting enzymes in the UDP-Gal module. The titer of LNnT increased from 0.76 g/L (BY04) to 1.01 g/L (BY14, overexpression of *pgcA* gene), 0.92 g/L (BY15, overexpression of *gtaB* gene), 1.0 g/L (BY16, knocking out *tuaD* gene), and 0.81 g/L (BY17, overexpression of *galE* gene). In the strain BY13 (overexpression of *pgi* gene in BY04), the titer of LNnT increased by 26.3% (0.96 g/L) compared with control strain BY04, whereas LNnT production decreased in the BY08 strain (overexpression of *pgi* gene in BY06), indicating that the Pgi was an effective enzyme in LNnT synthetic pathway. These results demonstrated that the insufficient supply of the two precursors was indeed the rate-limiting step in the synthesis of LNnT and also showed that the overexpression of the positive-acting enzymes in the biosynthetic pathways or deletion of the enzymes in the branching pathways would lead to the increase in production.

**Fig. 4:**
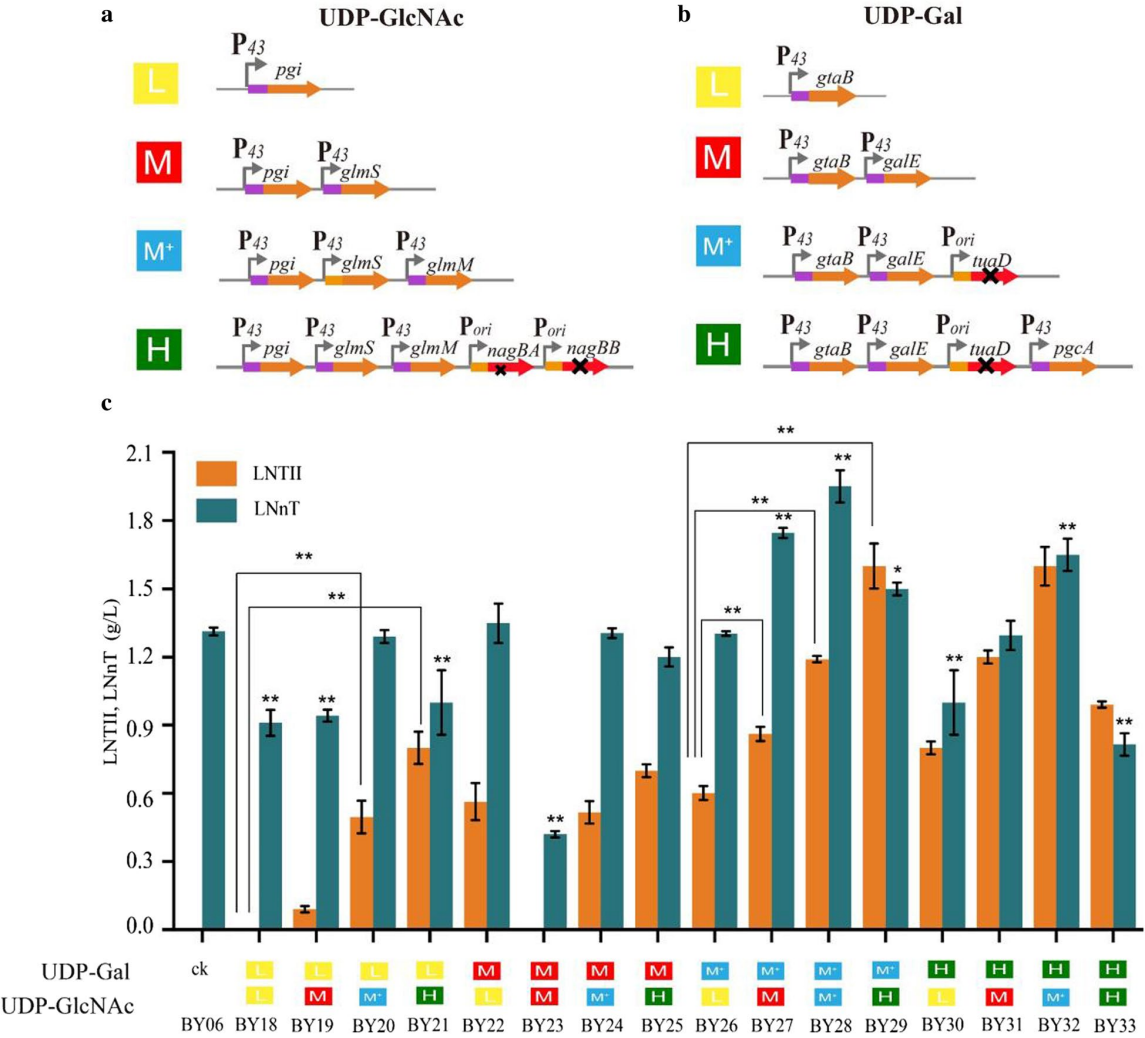
Effects of UDP-GlcNAc and UDP-Gal supply module on LNnT biosynthesis. **a** Four strengthen levels of UDP-GlcNAc module. **b** Four strengthen levels of UDP-Gal module. **c** Influences of modular engineering of LNnT and LNTII. Triplicate experiments were done, and error bars represent the standard deviation. * and** mean p < 0.05 and p < 0.01, respectively

The *pgi*, *glmS*, *glmM*, *nagBA*, and *nagBB* gene of UDP-GlcNAc pathway, and *pgcA*, *gtaB*, *tuaD,* and *galE* gene of UDP-Gal pathway were selected for modular engineering to improve the titer of LNnT. The UDP-GlcNAc module engineering was conducted into four different strength levels via combinatorial modulation of overexpression of *pgi*, *glmS*, *glmM* and co-deletion of *nagBA*, *nagBB* (Fig. 4a). Meanwhile, the UDP-Gal module engineering was also designed into four different levels of strength by assembling of overexpression of *pgcA*, *gtaB*, *galE,* and *tuaD* knockout (Fig. 4b). Next, the various strengths of the UDP-Gal and UDP-GlcNAc modules were assembled to obtain 16 strains, as shown in Fig. 4c.

### Effects of UDP-GlcNAc and UDP-Gal supply module on LNnT biosynthesis

The effects of various combinations of UDP-Gal and UDP-GlcNAc supply modules were compared in strain BY18–BY33 in shake-flask fermentation (Fig. 4c). When the UDP-Gal module was kept at a low level, the increased strength of the UDP-GlcNAc module from low to medium, medium-plus, and high level resulted in an increase in LNTII production to 0.1 g/L, 0.5 g/L, and 0.8 g/L (strains BY19, BY20, and BY21), comparing to below detection level (strain BY18). Similarly, when the UDP-Gal module was at a medium-plus level, the UDP-GlcNAc module increased from low to medium, medium-plus, and high level resulted in 43.3% (0.86 g/L), 98.3% (1.19 g/L), and 166.7% (1.6 g/L) increase in LNTII titer (strains BY27, BY28, and BY29). These results indicated that the supply of intracellular UDP-GlcNAc was deficient when UDP-GlcNAc module was kept at a low strength, and strengthen the level of UDP-GlcNAc module could effectively promote LNTII synthesis. Compared with controlling the UDP-GlcNAc module at medium-plus level and the UDP-Gal module at low level (1.29 g/L, strain BY20), the titer of LNnT increased by 2.3% and 51.2% (1.32 g/L, strain BY24and 1.95 g/L, strain BY28) when the UDP-Gal module level was increased to medium and medium-plus levels. The results indicated that the concentration of intracellular UDP-Gal was significantly low when UDP-Gal module was kept at a low strength.

As shown in Fig. 4c, improving the UDP-Gal module at medium-plus strength and the UDP-GlcNAc module at low level resulted in almost no increase in the titer of LNTII (0.6 g/L) and 3.8% (1.30 g/L) decrease in the titer of LNnT in the strain BY26, compared with the case when UDP-Gal module was at a medium level and the UDP-GlcNAc module still at low level with LNTII (0.56 g/L) and LNnT (1.35 g/L) in the strain BY22. On the basis of the strain BY26, the module strength of UDP-Gal was further increased to high level, which in turn resulted in a 23.1% decrease in LNnT titer (1.0 g/L, strain BY30). These results showed that UDP-Gal supply in strain BY26 was not a rate-limiting step, so when the UDP-Gal module was further strengthened, the titer was reduced due to the imbalance of the two modules. Conversely, when the module strength of UDP-GlcNAc was increased to medium strength based on strain BY26, LNTII titer increased by 43.3% (0.86 g/L) and LNnT titer increased by 34.6% (1.75 g/L) in the strain BY27. Strengthening the UDP-GlcNAc module led to a significant increase in titer, indicating that the balance of UDP-GlcNAc and UDP-Gal supply was critical to the efficient synthesis of LNnT, which was achieved through module engineering strategy.

**Fig. 5:**
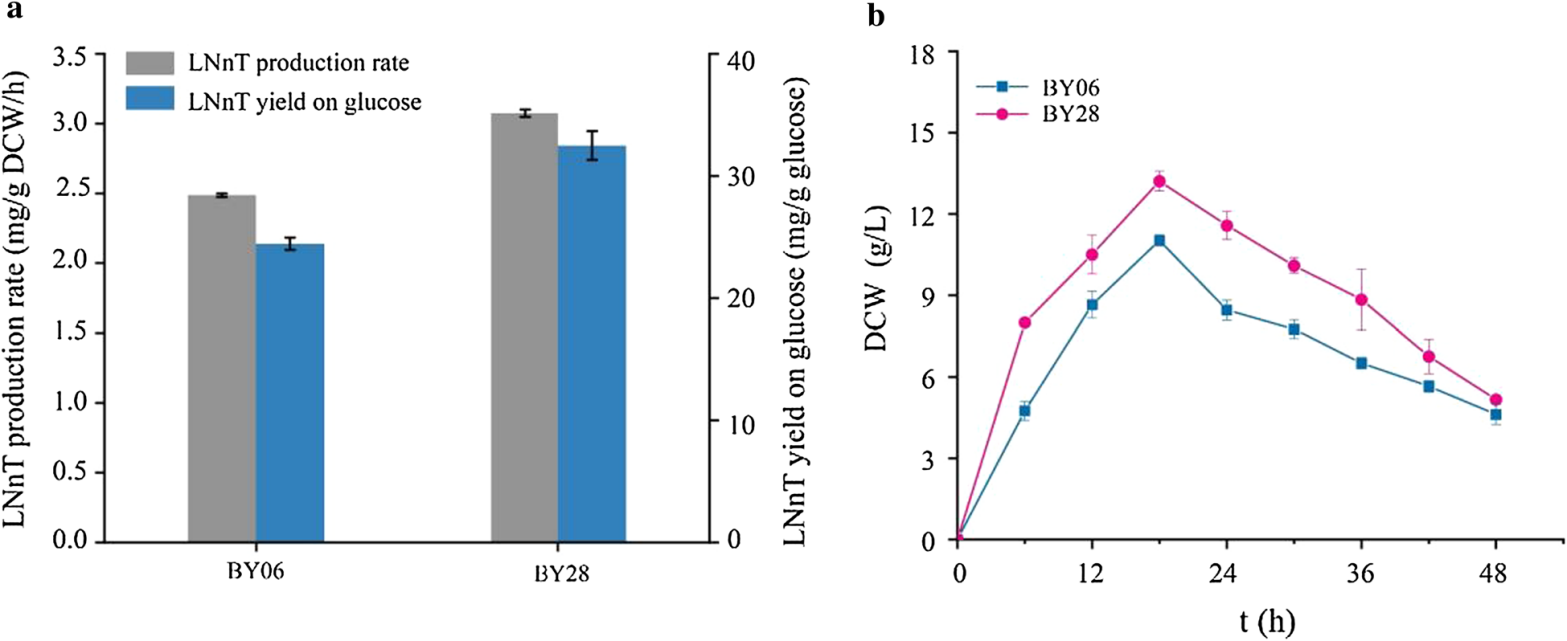
Effects of modular engineering on LNnT biosynthesis. **a** LNnT production rate, and yield on glucose in BY06 and BY28. **b** Comparison of cell growth of BY06 and BY28

In summary, compared with control strain BY06, the LNnT titer was significantly improved by both controlling the UDP-Gal and UDP-GlcNAc modules at medium-plus levels (strain BY28), the LNnT yield on glucose increased by 33.2% (32.5 mg/g glucose), and LNnT production rate increased by 23.3% (3.07 mg/g DCW/h), respectively, resulting in a maximal titer of 1.95 g/L LNnT at 48 h (Fig. 5a). After enhancing the synthetic pathway of the two precursors, the maximum DCW of strain BY28 increased to 13.2 g/L, which was 20% higher than that of strain BY06 (Fig. 5b). These results suggested that the supply and balance of the two precursors by module pathway engineering are critical for efficient production of LNnT. Noticeably, the accumulation of extracellular LNT II (1.19 g/L) in BY28 strain indicated that the catalytic ability of LgtB enzyme in BY28 may be insufficient. Unfortunately, increasing the expression level of LgtB may cause the pressure of cellular protein synthesis system to reduce LNnT synthesis efficiency. Therefore, improving the catalytic efficiency of key enzyme LgtB by engineering strategy will be an effective method to improve the synthesis efficiency of LNnT, such as enzyme protein engineering, enzyme evolution, and flow cytometry efficient screening, and phage-assisted continuous evolution [26, 27].

### Production of LNnT by BY28 in a 3-L bioreactor

**Fig. 6:**
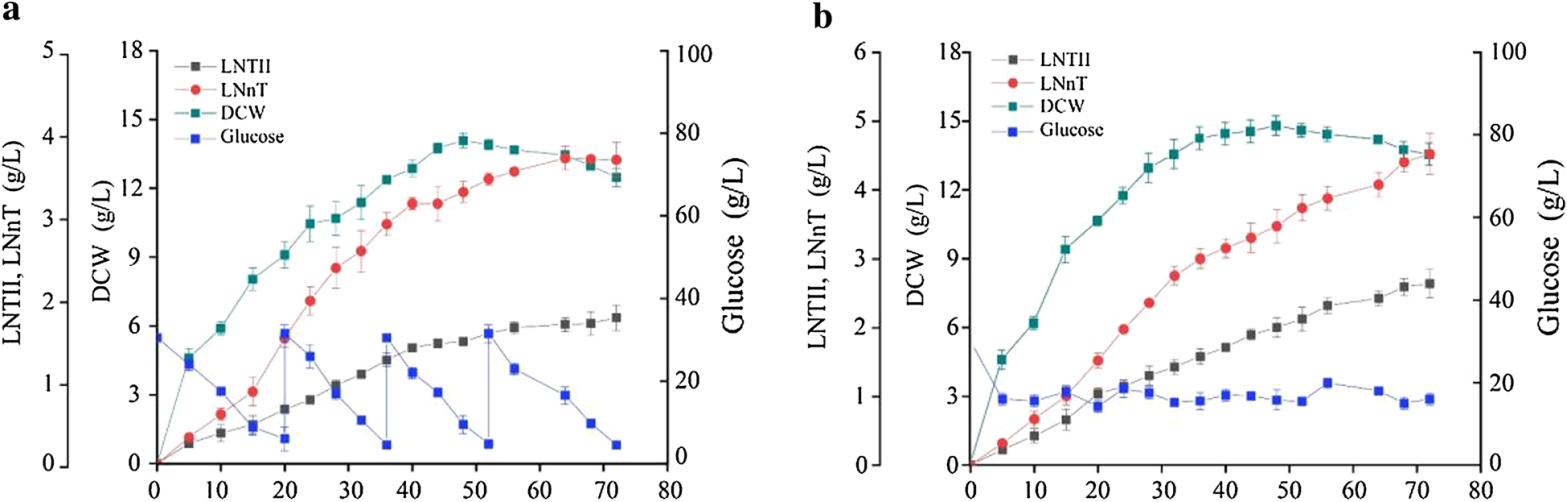
Production of LNnT by BY28 in a 3-L bioreactor. **a** Time course of the LNnT production in intermittent feeding fermentation. **b** Time course of the LNnT production in dual-flow continuous feeding fermentation. Triplicate experiments were done, and error bars represent the standard deviation

Based on the results of the modular engineering strategy, the engineered *B. subtilis* BY28 was used for LNnT production in a 3-L bioreactor by two feeding strategies. The LNnT titer with the time course under the intermittent feeding strategy and dual-flow continuous feeding strategy with control of glucose and lactose concentration is shown in Fig. 6a, b, respectively. In intermittent feeding fermentation, a total of 220 mL of glucose and lactose feeding solution was added to the 3-L fermentor during the 72 h of culture period, the maximum DCW was 14.5 ± 0.22 g/L, and the titer of LNnT and LNT II were 3.68 ± 0.15 g/L and 1.77 ± 0.11 g/L, respectively. The LNnT yield on glucose and the production rate was 40.4 ± 0.13 mg/g and 3.52 ± 0.061 mg/g DCW/h, respectively. In dual-flow continuous feeding fermentation, a total of 310 mL of glucose and lactose feeding solution was added to the 3-L fermentor during the 72 h of culture period, the maximum DCW was 14.7 ± 0.31 g/L, and the titer of LNnT and LNT II were 4.52 ± 0.21 g/L and 2.64 ± 0.15 g/L, respectively. The LNnT yield on glucose and the production rate were 41.9 ± 0.17 mg/g and 4.27 ± 0.055 mg/g DCW/h, respectively. These results showed that high concentrations of glucose and lactose in the fermentation broth result in reduced host synthesis efficiency of LNnT. Therefore, dual-flow continuous feeding strategy with control of glucose and lactose concentration was more suitable for LNnT production, and this might be useful for the scale-up of LNnT production. The total titer of LNnT (4.52 g/L) and LNTII (2.64 g/L) of BY28 was 7.16 g/L in the 3-L bioreactor, which was higher than the total titer of LNnT and its derivate (5 g/L) of *E. coli* JM109 in the 2-L reactor. Moreover, LNnT could be released in the extracellular medium by *B. subtilis*, but could not by *E. coli* JM109 [11]. Therefore, the *B. subtilis* was more suitable for industrial production of LNnT than *E. coli* JM109 because of convenient isolation.

## Conclusion

In this study, efficient synthesis of LNnT was achieved by optimizing the expression level of LgtA and LgtB and the modular optimization of two key precursors’ synthesis pathway. The overexpression of *glmM* or *tuaD* deletion was more useful to increase the production of LNnT than other genes in the synthesis of the two precursors, while the up-regulation of *glmU* was not suitable in the LNnT-producing strain. The titer of LNnT was improved step-by-step from 0.05 to 1.95 g/L in shake-flask culture, and 4.52 g/L in a 3-L bioreactor by feed batch. In summary, this study demonstrated that the optimization of heterologous proteins expression level and modular engineering of synthetic pathways are feasible and effective for improving the synthesis of LNnT in *B. subtilis*. This strategy could also be useful for producing other valuable oligosaccharides.

## Supplementary information

**Additional file 1.** Additional tables.

## Data Availability

The data supporting the results of the article are included in this manuscript and additional files.

## Abbreviations

LNnT: lacto-*N*-neotetraose
LNTII: Lacto-*N*-triose II
UDP-GlcNAc: UDP-*N*-acetyglucosamine
UDP-Gal: UDP-galactose
PEP: phosphoenolpyruvic acid
G6P: glucose-6-phosphate
F6P: fructose-6-phosphate
GlcN-6-P: glucosamine-6-phosphate
GlcN-1-P: glucosamine-1-phosphate
GlcNAc-1P: *N*-acetylglucosamine-1-phosphate
Glc-1P: glucose-1-phosphate
UDP-Glc: UDP-glucose
UDP-GlcA: UDP-glucuronate
DCW: dry cell weight
PCR: polymerase chain reaction
OD_600_: optical density at a wavelength of 600 nm
