# Supplementary material for: Modular pathway engineering of key precursor supply pathways for lacto-N-neotetraose production in Bacillus subtilis
Source: Biotechnol Biofuels. 2019 Sep 9;12:212. doi: 10.1186/s13068-019-1551-3 (PMC6732834; doi:10.1186/s13068-019-1551-3)
Supplement: Supplementary file 1 — Additional file 1. Additional tables. [file 13068_2019_1551_MOESM1_ESM.docx]

**Additional File 1**

**Table S1 Primers used in this study**

| Primer | Sequence |
| --- | --- |
| comk-L-F  comk-L-R  crm-PxylA-F  crm-PxylA-R  comk-F  comk-R  lacY-L-F  lacY-L-R  P7ZP43-lacY-F  P7ZP43-lacY-R  lacY-F  lacY-R  lacY-R-F  lacY-R-R  pP43-lgtA, B-1F  pP43-lgtA, B-1R  pP43-lgtA, B-2F  pP43-lgtA, B-2R  pP43-lgtA, B-3F  pP43-lgtA, B-3R  lgtA-L-F  lgtA-L-R  P7ZP43-lgtA-F  P7ZP43-lgtA-R  lgtA-F  lgtA-R  lgtA-R-F  lgtA-R-R  lgtB-L-F  lgtB-L-R  P7SP43-lgtB-F  P7SP43-lgtB-R  lgtB-F  lgtB-R  lgtB-R-F  lgtB-R-R  pP43-lgtA-1F  pP43-lgtA-1R  pP43-lgtA-2F  pP43-lgtA-2R  pHT01-lgtB-1F  pHT01-lgtB-1R  pHT01-lgtB-2F  pHT01-lgtB-2R  2lgtB-L-F  2lgtB-L-R  P7CP43-2lgtB-F  P7CP43-2lgtB-R  2lgtB-F  2lgtB-R  2lgtB-R-F  2lgtB-R-R  3lgtB-L-F  3lgtB-L-R  P7ZP43-3lgtB-F  P7ZP43-3lgtB-R  3lgtB-F  3lgtB-R  3lgtB-R-F  3lgtB-R-R  4lgtB-L-F  4lgtB-L-R  P7ZP43-4lgtB-F  P7ZP43-4lgtB-R  4lgtB-F  4lgtB-R  4lgtB-R-F  4lgtB-R-R  pgcA-L-F  pgcA-L-R  P7ZP43-pgcA-F  P7ZP43-pgcA-R  pgcA-F  pgcA-R  pgcA-R-F  pgcA-R-R  gtaB-L-F  gtaB-L-R  P7SP43-gtaB-F  P7SP43-gtaB-R  gtaB-F  gtaB-R  gtaB-R-F  gtaB-R-R  galE-L-F  galE-L-R  P7CP43-galE-F  P7CP43-galE-R  galE-F  galE-R  galE-R-F  galE-R-R  tuaD-L-F  tuaD-L-R  tuaD-F  tuaD-R  tuaD-R-F  tuaD-R-R  pgi-L-F  pgi-L-R  P7ZP43-pgi-F  P7ZP43-pgi-R  pgi-F  pgi-R  pgi-R-F  pgi-R-R  glmS-L-F  glmS-L-R  P7ZP43-glmS-F  P7ZP43-glmS-R  glmS-R-F  glmS-R-R  glmM-L-F  glmM-L-R  P7CP43-glmM-F  P7CP43-glmM-R  glmM-F  glmM-R  glmM-R-F  glmM-R-R  glmU-L-F  glmU-L-R  P7SP43-glmU-F  P7SP43-glmU-R  glmU-F  glmU-R  glmU-R-F  glmU-R-R  nagBA-L-F  nagBA-L-R  nagBA–F  nagBA-R  nagBA-R-F  nagBA-R-R  nagBB-L-F  nagBB-L-R  nagBB-F  nagBB-R  nagBB-R-F  nagBB-R-R | 5’-CGGTGTTATTCGCAAAAGAAGGGG-3’  5’-CGGGATCAAATCCGATGAAAGAGAAAAAATCGTACACTGAGCTCATG-3’  5’-CGATTTTTTCTCTTTCATCGGATTTGATCCCGTATACCGTT-3’  5’-CCTCCTTTAACATTTCCCCCTTTGATTTTTAGATATCACTAGTTTGGA-3’  5’-AATCAAAGGGGGAAATGTTAAAGGAGGAAGGATCCATGAGTCAGAAAACAGACG-3’  5’-GGCTATAATCCAAGCCCGATGCTGTTA-3’  5’-TATTCCGTATGTCAAGTGGCTGCGGTTTAT-3’  5’-AATTGTTATCCGCTCTCTTGACACTCCTTATTTGATTTTTTGAAGACTTACTTCGG-3’  5’-TCAAATAAGGAGTGTCAAGAGAGCGGATAACAATTTCACACAGGAAACAG-3’  5’-AGTTTGTGTTTTTTAAATAGTACATGTGTACATTCCTCTCTTACCTATAATGGTACCGC-3’  5’-GTAAGAGAGGAATGTACACATGTACTATTTAAAAAACACAAACTTTTGGATGTTCGGTT-3’  5’-TAAGTCCCGTCTAGCCTTGCCCTTAAGCGACTTCATTCACCTGACGACG-3’  5’-CTTAAGGGCAAGGCTAGACGGGACTTA-3’  5’-GGCACACCGATGTACACGTCATC-3’  5’-ATGCCGTCTGAAGCTTTTAGACGC-3’  5’-TTAGCGGTTTTTCAGCAGTCTATGCAGGATT-3’  5’-GACTGCTGAAAAACCGCTAACCCGGGGTAAGAGAGGAATGTACACATGCAAAATCATGTCATTTCTTTAGCATCAGCA-3’  5’-TTACTGAAACGGAACGATAAACTGTTCGC-3’  5’-TTATCGTTCCGTTTCAGTAATGATGAAAGCTTGGCGTAATCATGGTCATAG-3’  5’-CTAAAAGCTTCAGACGGCATGTGTACATTCCTCTCTTACCTATAATGGTACCGCTA-3’  5’-GTTGCCTGAGCACATACGGGACTAAACAA-3’  5’-CCTGTGTGAAATTGTTATCCGCTCTCAATCGTCTTTGCGAAGTCCGAGTCCAAGTT-3’  5’-GACGATTGAGAGCGGATAACAATTTCACACAGGAAACAG-3’  5’-AAGCTTCAGACGGCATGTGTACATTCCTCTCTTACCTATAATGGTACCGC-3’  5’-GTAAGAGAGGAATGTACACATGCCGTCTGAAGCTTTTAGACGC-3’  5’-AAAAAGCCCGCTCATTAGGCGGGCTTCATCATTAGCGGTTTTTCAGCAGTCTATGCAGG-3’  5’-GCCCGCCTAATGAGCGGGCTTTTTTCTAGTTTCCCTTGTGAACTAGGATTTTCGTGT-3’  5’-CGGCTCTTGATCGAGAAAATCTTGCATATTCA-3’  5’-GGGACAAGGAATAGTAAGCCGGCAA-3’  5’-TCCTGTGTGAAATTGTTATCCGCTCCTACATACTCTCTGTAGCAGAGGTAGCTTGA-3’  5’-AGAGTATGTAGGAGCGGATAACAATTTCACACAGGA-3’  5’-AAAGAAATGACATGATTTTGCATGTGTACATTCCTCTCTTACCTATAATGGTACCGC-3’  5’-AGGTAAGAGAGGAATGTACACATGCAAAATCATGTCATTTCTTTAGCATCAGCAG-3’  5’-CCCGCTCATTAGGCGGGCTTCATCATTACTGAAACGGAACGATAAACTGTTCGCG-3’  5’-TGAAGCCCGCCTAATGAGCGGGCTTTTTTCTGATAAGAACTGCAAAAGCTGCGGATTAT-3’  5’-CCACCCTATAGATAAATTTTTCGGCTGCCATAT-3’  5’-ATGCCGTCTGAAGCTTTTAGACGC-3’  5’-TTAGCGGTTTTTCAGCAGTCTATGCAGGA-3’  5’-GACTGCTGAAAAACCGCTAATGATGAAAGCTTGGCGTAATCATGGTC-3’  5’-CTAAAAGCTTCAGACGGCATGTGTACATTCCTCTCTTACCTATAATGGTACCGCT-3’  5’-AAAGGAGGTGAAATGTACACATGCAAAATCATGTCATTTCTTTAGCATCAGCAG-3’  5’-TTACTGAAACGGAACGATAAACTGTTCGCG-3’  5’-GTGTACATTTCACCTCCTTTAAATTACTTTCATTATGAGTTAAATTTCC-3’  5’-TTATCGTTCCGTTTCAGTAATCTAGAGTCGACGTCCCCGGG-3’  5’-CATCCAGCGTAAAACGTTCACGGGAATAATCTAGG-3’  5’-TTATCCGCTCTGGCGCGGACTTGTTTGTTTATATCCATTCTAAATGAAGG-3’  5’-ACAAGTCCGCGCCAGAGCGGATAACAATTTCACACAGGAAACAGCTATG-3’  5’-AGAAATGACATGATTTTGCATGTGTACATTCCTCTCTTACCTATAATGGTACCGCT-3’  5’-ATAGGTAAGAGAGGAATGTACACATGCAAAATCATGTCATTTCTTTAGCATCAGCAGCG-3’  5’-GCCCGCTCATTAGGCGGGCTTCATCATTACTGAAACGGAACGATAAACTGTTCGCGT-3’  5’-GCCCGCCTAATGAGCGGGCTTTTTTTTAAGACGTGGACTCGTTTTCAGCCTGAAATTTT-3’  5’-CATCCCAGCAGCTGATCAGGATGAATTCT-3’  5’-GGCTTTCGTTGTCTTGTGTTCAAGAAATTTCCA-3’  5’-CTGTGTGAAATTGTTATCCGCTCCCAGCCTTTCTTATTAAAACCACTTTGTCAGCC-3’  5’-GTTTTAATAAGAAAGGCTGGGAGCGGATAACAATTTCACACAGGAAACAGC-3’  5’-AAAGAAATGACATGATTTTGCATGTGTACATTCCTCTCTTACCTATAATGGTACCGC-3’  5’-AGGTAAGAGAGGAATGTACACATGCAAAATCATGTCATTTCTTTAGCATCAGCAG-3’  5’-CCCGCTCATTAGGCGGGCTTCATCATTACTGAAACGGAACGATAAACTGTTCGCG-3’  5’-AAGCCCGCCTAATGAGCGGGCTTTTTTATCATAAAGATCCAGCCTTTTTGCGCT-3’  5’-CTAAAGCCCAAGTCACAATATATTGATCGCCT-3’  5’-TTGCGGTACAAGTTGTGGCAAATTCCTACGGA-3’  5’-CGCAATAACGCAGGCGTTCTGTGACATTAACTTATTTCATTAAGCAGCGTTTTTTGTTTGTCTCAGCGTGCG-3’  5’-  GTCACAGAACGCCTGCGTTATTGCGCAGGCGTTTTGTAATAAAGAGCGGATAACAATTTCACACAGGAAACAGCT-3’  5’-AAAGAAATGACATGATTTTGCATGTGTACATTCCTCTCTTACCTATAATGGTACCGC-3’  5’-AGGTAAGAGAGGAATGTACACATGCAAAATCATGTCATTTCTTTAGCATCAGCAG-3’  5’-GGGATTTCCCGGCAGTCTGACAAGTTATTCTGCAATAGACACTTTTCCTTACTGAAACGGAACGATAAACTGTTCGCG-3’  5’-GAATAACTTGTCAGACTGCCGGGAAATCCCGGCAGTCTTTTTTCCATTTTTGATCATCTCAAAAGCGTTTTTTTATCTGATTTATTGGT-3’  5’-TCGTCTCGGCTGAAGCCATCACAATCG-3’  5’-AGCTTCTGCTTTTCCATGTACTCCCCCCTCT-3’  5’-TCCTGTGTGAAATTGTTATCCGCTCAAAAAGGTGCTCCTTACGGCCAAATCTCATCCGC-3’  5’-CCGTAAGGAGCACCTTTTTGAGCGGATAACAATTTCACACAGGAAACAGCTATGACCAT-3’  5’-AGCGTTCATAGCTCTTTCTCCAAGTCATGTGTACATTCCTCTCTTACCTATAATGGTACCGCTATCAC-3’  5’-GAGAGGAATGTACACATGACTTGGAGAAAGAGCTATGAACGCTGGAAACAGACAGA-3’  5’-GAAAAAAGCCCGCTCATTAGGCGGGCTTTATTTTGCTGTTGACTCAACAATTTCATCCACCGTCTTCATTACATCTTCAG-3’  5’-AGCCCGCCTAATGAGCGGGCTTTTTTCTAATACACAGCCTCTTCCGTTTCCGCATCGAAAAAGACG-3’  5’-CGTTTTCGGTAGGCAGCTTGGCGGTTGGGGAATAC-3’  5’-GGCAACGGTTCATCTTTTTCTCAGAACG-3’  5’-CCTGTGTGAAATTGTTATCCGCTCTTATTCCACGAGATAAAGAGTTTGCTGGCT-3’  5’-CTTTATCTCGTGGAATAAGAGCGGATAACAATTTCACACAGGAAACAG-3’  5’-TGGCTTTACGTACTTTTTTCATGTGTACATTCCTCTCTTACCTATAATGGTACCGC-3’  5’-AGGTAAGAGAGGAATGTACACATGAAAAAAGTACGTAAAGCCATAATTCCAGCA-3’  5’-GCCCGCTCATTAGGCGGGCTTTAGATTTCTTCTTTGTTTAGTAAACCTTCCATAAATGGAACG-3’  5’-GCCCGCCTAATGAGCGGGCTTTTTTTTAAGCTTCTTCTTTTACATGTACCTTTAACACAGCCTGC-3’  5’-CCATCACCGAAGAGGTGAGGAGTTTATCAGAGAT-3’  5’-GTGAAGCGGCGTATTATTAAAGGAAAGGCTGTA-3’  5’-TGTGTGAAATTGTTATCCGCTCTCACACTTCCACTGTCCAGTTAAACGGGC-3’  5’-CAGTGGAAGTGTGAGAGCGGATAACAATTTCACACAGGAAACAG-3’  5’- GCCAGTAACAAGTATTGCCATGTGTACATTCCTCTCTTACCTATAATGGTACCGC-3’  5’-AAGAGAGGAATGTACACATGGCAATACTTGTTACTGGCGGT-3’  5’-CCCGCTCATTAGGCGGGCTTTATTCCGCACTCTTATACCCATTCACATTAGAAGA-3’  5’-AGCCCGCCTAATGAGCGGGCTTTTTTAAATCGAAAAAGAACCTGCCCGGAGGCA-3’  5’-CCGGATTTACGGTCGCTTGGCTGTATT-3’  5’-CAAATTCCTTATGTCCTGACCTTGCATGGC-3’  5’-TGTGTGAAATTGTTATCCGCTCAGCGCTCAACCCTCTCCTGTAAAAT-3’  5’-GTTGAGCGCTGAGCGGATAACAATTTCACACAGGAAACAGCT-3’  5’-GAGGGTTCCGTAACGCCAGGGTTTTCCCAGTCACGAC-3’  5’-ACCCTGGCGTTACGGAACCCTCTGACAAGTATTTTCCGG-3’  5’-GCCATTCTCTTTAAAACAGGCGGCAATACAA-3’  5’-GAGGGTACAAAGATGTGAACTATACGCTTGC-3’  5’-GAAATTGTTATCCGCTCAAGGCCTTAAGCCTTATACCTTTTTTAATTTTAAGTCCGA-3’  5’-GGTATAAGGCTTAAGGCCTTGAGCGGATAACAATTTCACACAGGAAACAG-3’  5’-CAAAGCGTACATGCGTCATGTGTACATTCCTCTCTTACCTATAATGGTACCGC-3’  5’-AGAGGAATGTACACATGACGCATGTACGCTTTGACTACTCAAAA-3’  5’-GCCCGCTCATTAGGCGGGCTTCATCATTAATCTTCCAGACGTTTTTCAAGCTCTGCTT-3’  5’-GCCCGCCTAATGAGCGGGCTTTTTTATACCTTTTTGATTAATCATTCCATTGATACGTCCA-3’  5’-CATGAAAGCGATCATAAAAGAAGATGTGCAAG-3’  5’-CCAGGTGTATCTTATTTGACAAAAGCGATGGATGCA-3’  5’-GTTATCCGCTCTTACTCTAATCCCATTTCTGACCGGACGACTTCAACAAT-3’  5’-CCGGTCAGAAATGGGATTAGAGTAAGAGCGGATAACAATTTCACACAGGAAACAG-3’  5’-CCTACGATTCCACACATGTGTACATTCCTCTCTTACCTATAATGGTACCGC-3’  5’-GGTAAGAGAGGAATGTACACATGTGTGGAATCGTAGGTTATATCGGTCAGCTTGAT-3’  5’-GTCTGCTGTTTCTCCGCTTTGAGAAAGGAAAATGA -3’  5’-GGACAGGTTTTCTGTTTGAATGAAGCCGT-3’  5’-GTGTGAAATTGTTATCCGCTCCAGCAGGAGCTATACATAGCAAACAAAAAAGAGC-3’  5’-GCTCCTGCTGGAGCGGATAACAATTTCACACAGGAAACAG-3’  5’-CTGTTCCAAAATACTTGCCCATGTGTACATTCCTCTCTTACCTATAATGGTACCGC-3’  5’-GGTAAGAGAGGAATGTACACATGGGCAAGTATTTTGGAACAGACGG-3’  5’- GCCCGCTCATTAGGCGGGCTGCCCCGGGTTACTCTAATCCCATTTCTGACCGGACGAC-3’  5’-CAGCCCGCCTAATGAGCGGGCTTTTTTCACGTCTTATTCAGCAGCAGAAACTTCTCTGATCGC-3’  5’-GATCCAAAACGCACTCGGCTCTGATATT-3’  5’-GCGAACAGGCATCCTATACACTGGGAC-3’  5’-CTGTGTGAAATTGTTATCCGCTCTTATTAGACGGAGTCTTTTTTGCTTTTGCCAATCAG-3’  5’-AGACTCCGTCTAATAAGAGCGGATAACAATTTCACACAGGAAACAG-3’  5’-CTGCAAACCGCTTATCCATGTGTACATTCCTCTCTTACCTATAATGGTACCGC-3’  5’-GAGGAATGTACACATGGATAAGCGGTTTGCAGTTGTTTTAGCG-3’  5’-GACTGCCGGGATTTCCCGGCAGTCTGACAAGTTATTTATTTTTTATGAATATTTTTCACATAATCGTCTTTATTTACTTGTCTCGCTCT-3’  5’-GCCGGGAAATCCCGGCAGTCTTTTTTCCATTGACGGAGTCTTTTTTTATTTCGTTTTTAAGAAGTAGGT-3’  5’-CTAACACAATCCATTTTGAAGATGCCTTTTTGCA-3’  5’-ATGGCAGAGAGTCTTCTTATCAAAGACATTGC-3’  5’-TGTGAAATTGTTATCCGCTCTCATATCTGGTCAGCCTCCTTGGATATAAATGCA-3’  5’-ATCCAAGGAGGCTGACCAGATATGAGAGCGGATAACAATTTCACACAGGAAACAGCT-3’  5’-TCCTTCTAACAGCTTACGAACAGTAACGCCAGGGTTTTCCCAGTCACGAC-3’  5’-CTGGGAAAACCCTGGCGTTACTGTTCGTAAGCTGTTAGAAGGAAACATAAGCGA-3’  5’- AGAACTACTATGATACGGATACCTATGGCAACAAG-3’  5’-TGCGAATACAAATTGCCAATTGATCTTCCGC-3’  5’-TCCTGTGTGAAATTGTTATCCGCTCGTGACACCCCCTCAAAGAGATAGACAAG-3’  5’-TCTCTTTGAGGGGGTGTCACGAGCGGATAACAATTTCACACAGGAAACAGCT-3’  5’-TTTGAAGAATGGAGGCTGGTACTAACGCCAGGGTTTTCCCAGTCACGAC-3’  5’-ACTGGGAAAACCCTGGCGTTAGTACCAGCCTCCATTCTTCAAAAACACAAT-3’  5’-AGTCCGTAGTTCAAGACGTAGTCAATTCC-3’ |
|  |  |

Underlined letters represent homologous sequences for fusion PCR

**Table S2 Sequences of codon-optimized *lgtA* gene**

| **Name** | **Sequence (5’→3’)** |
| --- | --- |
| *lgtA* | ATGCCGTCTGAAGCTTTTAGACGCCATAGAGCCTATCGCGAAAATAAACTTCAACCGTTAGTCTCTGTTTTAATCTGCGCTTATAACGTGGAAAAATATTTTGCCCAATCACTGGCAGCGGTTGTGAATCAGACATGGAGAAACCTGGATATTCTTATCGTCGATGATGGCTCAACAGATGGAACACTTGCTATTGCCCAAAGATTTCAAGAACAGGATGGCAGAATTCGCATCTTAGCACAGCCGCGCAATTCTGGCCTTATTCCGTCATTAAACATCGGATTAGATGAACTGGCGAAAAGCGGCGGAGGCGGAGAATATATCGCTAGAACAGATGCCGATGATATTGCTGCCCCGGATTGGATTGAAAAAATCGTTGGCGAAATGGAAAAAGATAGATCAATCATCGCAATGGGAGCGTGGCTTGAAGTGTTAAGCGAAGAAAAAGATGGCAATAGACTGGCTCGCCATCATGAACATGGAAAAATCTGGAAAAAACCGACAAGACATGAAGATATTGCCGATTTCTTTCCGTTTGGCAATCCGATTCATAATAACACAATGATCATGAGACGCAGCGTCATTGATGGCGGACTTAGATATAACACAGAACGCGATTGGGCGGAAGATTACCAGTTTTGGTACGATGTTTCTAAACTGGGAAGACTTGCATATTATCCGGAAGCGCTGGTGAAATATCGCCTTCATGCTAATCAAGTCTCAAGCAAATATAGCATCAGACAGCATGAAATTGCACAAGGCATCCAGAAAACAGCGCGCAACGATTTTCTTCAAAGCATGGGATTTAAAACAAGATTTGATTCTCTGGAATACCGCCAGATCAAAGCAGTTGCGTATGAACTGCTTGAAAAACATCTGCCGGAAGAAGATTTTGAAAGAGCAAGACGCTTTCTTTACCAATGTTTTAAACGCACAGATACATTACCGGCTGGCGCCTGGCTGGATTTTGCAGCGGATGGAAGAATGAGACGCTTATTTACACTGCGCCAGTACTTTGGAATCCTGCATAGACTGCTGAAAAACCGCTAA |

**Table S3 Sequences of codon-optimized *lgtB* gene**

| **Name** | **Sequence (5’→3’)** |
| --- | --- |
| *lgtB* | ATGCAAAATCATGTCATTTCTTTAGCATCAGCAGCGGAAAGACGCGCTCATATTGCCGATACATTTGGCAGACATGGAATCCCGTTTCAATTTTTCGATGCGCTTATGCCGTCAGAACGCTTAGAACAGGCAATGGCGGAATTAGTTCCGGGCCTGTCAGCTCATCCGTATCTTAGCGGAGTGGAAAAAGCATGCTTTATGAGCCATGCGGTCTTATGGAAACAAGCTCTTGATGAAGGCCTGCCGTACATCACAGTTTTTGAAGATGATGTGCTGCTTGGCGAAGGAGCCGAAAAATTTCTGGCAGAAGATGCGTGGCTTCAGGAAAGATTTGATCCGGATACAGCATTTATCGTGCGCTTAGAAACAATGTTTATGCATGTCCTGACATCACCGAGCGGCGTTGCCGATTATTGTGGAAGAGCATTTCCGTTACTGGAATCTGAACATTGGGGCACAGCGGGATACATCATCTCAAGAAAAGCTATGAGATTTTTCCTGGATAGATTTGCTGCCCTTCCGCCGGAAGGCTTACATCCGGTTGATCTGATGATGTTTTCTGATTTCTTTGATCGCGAAGGAATGCCGGTGTGCCAACTGAATCCGGCTCTTTGTGCCCAGGAACTTCATTACGCCAAATTTCATGATCAAAACAGCGCACTGGGATCTCTTATCGAACATGATAGACTTCTGAACCGCAAACAACAGAGACGCGATAGCCCGGCGAACACATTTAAACATAGATTAATTCGCGCTCTGACAAAAATCTCTAGAGAACGCGAAAAAAGACGCCAAAGACGCGAACAGTTTATCGTTCCGTTTCAGTAA |

**Table S4 Genomic locus of *lgtA* gene integration expression**

| **Name** | **Content (5’→3’)** |
| --- | --- |
| left homology arm | GTTGCCTGAGCACATACGGGACTAAACAATGGGGAATTTCGGATGGAATCTCATATGATTCTATTCGAGGTTTCGACGTTTTGAAGGAGGGTTTTAAGTAATGATCGAGATTGAAAAACCAAAAATCGAAACGGTTGAAATCAGCGACGATGCCAAATTTGGTAAGTTTGTCGTAGAGCCACTTGAGCGTGGATATGGTACAACTCTGGGTAACTCCTTACGTCGTATCCTCTTATCCTCACTCCCTGGTGCCGCTGTAACATCAATCCAGATAGATGGTGTACTGCACGAATTCTCGACAATTGAAGGCGTTGTGGAAGATGTTACAACGATTATCTTACACATTAAAAAGCTTGCATTGAAAATCTACTCTGATGAAGAGAAGACGCTAGAAATTGATGTACAGGGTGAAGGAACTGTAACGGCAGCTGATATTACACACGATAGTGATGTAGAGATCTTAAATCCTGATCTTCATATCGCGACTCTTGGTGAGAATGCGAGTTTCCGAGTTCGCCTTACTGCTCAAAGAGGACGTGGGTATACGCCTGCTGACGCAAACAAGAGAGACGATCAGCCAATCGGCGTGATTCCGATCGATTCTATCTATACGCCAGTTTCTCGTGTATCTTATCAGGTAGAGAACACTCGTGTAGGCCAAGTTGCAAACTATGATAAACTTACACTTGATGTTTGGACTGATGGAAGCACTGGACCGAAAGAAGCAATTGCGCTTGGTTCAAAGATTTTAACTGAACACCTTAATATATTCGTTGGTTTAACTGACGAAGCTCAACATGCTGAAATCATGGTTGAAAAAGAAGAAGATCAAAAAGAGAAAGTTCTTGAAATGACAATTGAAGAATTGGATCTTTCTGTTCGTTCTTACAACTGCTTAAAGCGTGCGGGTATTAACACGGTTCAAGAGCTTGCGAACAAGACGGAAGAAGATATGATGAAAGTTCGAAATCTAGGACGCAAATCACTTGAAGAAGTGAAAGCGAAACTAGAAGAACTTGGACTCGGACTTCGCAAAGACGATTGA |
| Zeocin resistance and P*_43_* promoter | GAGCGGATAACAATTTCACACAGGAAACAGCTATGACCATGATTACGAATTCGAGCTCGGTACCCGGGGATCCTCTAGAGATACCGTTCGTATAGCATACATTATACGAAGTTATCTTGATATGGCTTTTTATATGTGTTACTCTACATACAGAAAGGAGGAACTAAACATGGCCAAGTTGACCAGTGCCGTTCCGGTGCTCACCGCGCGCGACGTCGCCGGAGCGGTCGAGTTCTGGACCGACCGGCTCGGGTTCTCCCGGGACTTCGTGGAGGACGACTTCGCCGGTGTGGTCCGGGACGACGTGACCCTGTTCATCAGCGCGGTCCAGGACCAGGTGGTGCCGGACAACACCCTGGCCTGGGTGTGGGTGCGCGGCCTGGACGAGCTGTACGCCGAGTGGTCGGAGGTCGTGTCCACGAACTTCCGGGACGCCTCCGGGCCGGCCATGACCGAGATCGGCGAGCAGCCGTGGGGGCGGGAGTTCGCCCTGCGCGACCCGGCCGGCAACTGCGTGCACTTCGTGGCCGAGGAGCAGGACTGAATAACTTCGTATAGCATACATTATACGAACGGTAAATCGTCGACTGATAGGTGGTATGTTTTCGCTTGAACTTTTAAATACAGCCATTGAACATACGGTTGATTTAATAACTGACAAACATCACCCTCTTGCTAAAGCGGCCAAGGACGCCGCCGCCGGGGCTGTTTGCGTTCTTGCCGTGATTTCGTGTACCATTGGTTTACTTATTTTTTTGCCAAGGCTGTAATGGCTGAAAATTCTTACATTTATTTTACATTTTTAGAAATGGGCGTGAAAAAAAGCGCGCGATTATGTAAAATATAAAGTGATAGCGGTACCATTATAGGTAAGAGAGGAATGTACAC |
| *lgtA* gene | ATGCCGTCTGAAGCTTTTAGACGCCATAGAGCCTATCGCGAAAATAAACTTCAACCGTTAGTCTCTGTTTTAATCTGCGCTTATAACGTGGAAAAATATTTTGCCCAATCACTGGCAGCGGTTGTGAATCAGACATGGAGAAACCTGGATATTCTTATCGTCGATGATGGCTCAACAGATGGAACACTTGCTATTGCCCAAAGATTTCAAGAACAGGATGGCAGAATTCGCATCTTAGCACAGCCGCGCAATTCTGGCCTTATTCCGTCATTAAACATCGGATTAGATGAACTGGCGAAAAGCGGCGGAGGCGGAGAATATATCGCTAGAACAGATGCCGATGATATTGCTGCCCCGGATTGGATTGAAAAAATCGTTGGCGAAATGGAAAAAGATAGATCAATCATCGCAATGGGAGCGTGGCTTGAAGTGTTAAGCGAAGAAAAAGATGGCAATAGACTGGCTCGCCATCATGAACATGGAAAAATCTGGAAAAAACCGACAAGACATGAAGATATTGCCGATTTCTTTCCGTTTGGCAATCCGATTCATAATAACACAATGATCATGAGACGCAGCGTCATTGATGGCGGACTTAGATATAACACAGAACGCGATTGGGCGGAAGATTACCAGTTTTGGTACGATGTTTCTAAACTGGGAAGACTTGCATATTATCCGGAAGCGCTGGTGAAATATCGCCTTCATGCTAATCAAGTCTCAAGCAAATATAGCATCAGACAGCATGAAATTGCACAAGGCATCCAGAAAACAGCGCGCAACGATTTTCTTCAAAGCATGGGATTTAAAACAAGATTTGATTCTCTGGAATACCGCCAGATCAAAGCAGTTGCGTATGAACTGCTTGAAAAACATCTGCCGGAAGAAGATTTTGAAAGAGCAAGACGCTTTCTTTACCAATGTTTTAAACGCACAGATACATTACCGGCTGGCGCCTGGCTGGATTTTGCAGCGGATGGAAGAATGAGACGCTTATTTACACTGCGCCAGTACTTTGGAATCCTGCATAGACTGCTGAAAAACCGCTAA |
| right homology arm | CTAGTTTCCCTTGTGAACTAGGATTTTCGTGTGTTTATTATTCAGAAACAGCATTCCAATAAAGGAGGGGACATCACATGTCATACAGAAAACTAGGACGTACGAGTGCACAGCGTAAAGCTATGCTTCGTGATCTTACAACTGATTTGATCATCAACGAAAGAATCGAAACAACTGAAACACGTGCGAAAGAACTTCGCTCTGTAGTTGAAAAAATGATCACGCTTGGCAAACGCGGTGATCTTCACGCTCGCCGTCAAGCTGCTGCATACATCCGCAACGAGGTTGCAAACGAAGAAAATAATCAAGATGCACTTCAAAAATTATTCTCTGACATTGCAACTCGTTACGAGGAGCGCCAAGGTGGATACACACGTATTATGAAGCTTGGTCCTCGCCGTGGTGACGGAGCACCAATGGCAATTATCGAATTGGTTTAATCACATATTTTTGTGTATCTAAAGAAGGGCGGGACAGTTTCTAACTGGATCTATGCCCTTTTTTTAGATACTGCAACTTTTAATAGAGAGAAAAGCAGAGCATTAGCTGAGAGGAGGCCGGTTTTCCATGAATCAAAATCAGTTGATATCGGTAGAGGATATCGTATTTCGATATCGGAAGGACGCAGAAAGACGAGCACTAGACGGCGTCTCCCTGCAGGTGTATGAGGGTGAATGGCTTGCAATCGTAGGTCATAACGGTTCAGGGAAATCAACACTGGCCCGGGCATTGAATGGTTTAATTCTTCCTGAATCAGGCGACATTGAGGTTGCCGGGATTCAATTGACAGAGGAATCTGTTTGGGAAGTGCGTAAGAAGATAGGTATGGTCTTTCAAAATCCGGATAACCAATTTGTCGGAACGACTGTTCGCGATGATGTGGCTTTTGGTTTAGAAAACAATGGTGTACCGCGGGAAGAAATGATTGAGAGAGTAGACTGGGCAGTAAAACAGGTGAATATGCAAGATTTTCTCGATCAAGAGCCG |

**Table S5 Genomic locus of 1 *lgtB* gene integration expression**

| **Name** | **Content (5’→3’)** |
| --- | --- |
| Left homology arm | GGGACAAGGAATAGTAAGCCGGCAACGTATCCGATTTGGGTGAGTGTGACAATCAATCCAGCACCGCTTGGAGAAAGTCCAATTGCCTTACTAATTAATCCGACCAAGGGTTGTGCATAGTAAAGATTAGCAACAATGATACCGCATGCAGTTGCGAGAAGAAGGGTTAAACTGGGTGAAATCCTCTGACCTGCTTGTTTGTTTCCATTAGCCATATGAATGAACTCCTTTTTAAGTGGTTTTTTGAGTGTAATCATGACTTCTCACAAGTGATTTTATAAACTGAACGTTCAGTTTCGTCGATTAATAAAAATAATATACTAAACGTTCAGTTTTGTAAATGGATTTCTGTTTTCTTTTTTTATTCAATTTAAGTATACTGAACGTATAGTTTATATTGCGGGAAGGAATGATTTATGTGCAGAGTAAACGAGGGCGGCCGCGTGATGAAGGAACGCATAAGGCGATTCTCTCTGCAGCCTATGACCTATTGCTGGAAAAAGGCTTCGATGCGGTGACAGTCGATAAAATTGCCGAGCGTGCGAAAGTGAGTAAAGCAACGATTTATAAATGGTGGTCTAACAAGGCTGCCGTTATCATGGACAGCTTTCTTTCGACCGCGACGGACAGGCTGCCTGTGCCTGATACAGGGTCGTCAGTACAAGATATAGTAACCCACGCCACGAATTTAGCTAGGTTTTTGACAAGCCGGGAAGGAACCGTTATTAAGGAATTAATAGGTGCAGGGCAGTTGGATGAAAAATTGGCAGAAGAATATCGCACGCGATTTTTCCAGCCTCGCCGCCTCCAAGCGAAAGGCCTTCTAGAAAAGGGAATTCAGAAAGGTGAATTGAGAGAGAATCTTGATATTGAAGTAAGCATAGATCTCATTTACGGACCAATTTTCTATCGTCTGCTTATAACAGGGGATGAGGTGAATGATTCCTATGTGCGTGATTTGGTGATGAATGCGTTTAAGGGAGTTCAAGCTACCTCTGCTACAGAGAGTATGTAG |
| Spectinomycin resistance and P*_43_* promoter | GAGCGGATAACAATTTCACACAGGAAACAGCTATGACCATGATTACGAATTCGAGCTCGGTACCCGGGGATCCTCTAGAGATTGTACCGTTCGTATAGCATACATTATACGAAGTTATCGATTTTCGTTCGTGAATACATGTTATAATAACTATAACTAATAACGTAACGTGACTGGCAAGAGATATTTTTAAAACAATGAATAGGTTTACACTTACTTTAGTTTTATGGAAATGAAAGATCATATCATATATAATCTAGAATAAAATTAACTAAAATAATTATTATCTAGATAAAAAATTTAGAAGCCAATGAAATCTATAAATAAACTAAATTAAGTTTATTTAATTAACAACTATGGATATAAAATAGGTACTAATCAAAATAGTGAGGAGGATATATTTGAATACATACGAACAAGTTAATAAAGTGAAAAAAATACTTCGGAAACATTTAAAAAATAACCTTATTGGTACTTACATGTTTGGATCAGGAGTTGAGAGTGGACTAAAACCAAATAGTGATCTTGACTTTTTAGTCGTCGTATCTGAACCATTGACAGATCAAAGTAAAGAAATACTTATACAAAAAATTAGACCTATTTCAAAAAAAATAGGAGATAAAAGCAACTTACGATATATTGAATTAACAATTATTATTCAGCAAGAAATGGTACCGTGGAATCATCCTCCCAAACAAGAATTTATTTATGGAGAATGGTTACAAGAGCTTTATGAACAAGGATACATTCCTCAGAAGGAATTAAATTCAGATTTAACCATAATGCTTTACCAAGCAAAACGAAAAAATAAAAGAATATACGGAAATTATGACTTAGAGGAATTACTACCTGATATTCCATTTTCTGATGTGAGAAGAGCCATTATGGATTCGTCAGAGGAATTAATAGATAATTATCAGGATGATGAAACCAACTCTATATTAACTTTATGCCGTATGATTTTAACTATGGACACGGGTAAAATCATACCAAAAGATATTGCGGGAAATGCAGTGGCTGAATCTTCTCCATTAGAACATAGGGAGAGAATTTTGTTAGCAGTTCGTAGTTATCTTGGAGAGAATATTGAATGGACTAATGAAAATGTAAATTTAACTATAAACTATTTAAATAACAGATTAAAAAAATTATAAATAACTTCGTATAGCATACATTATACGAACGGTAGAATCGTCGACTGATAGGTGGTATGTTTTCGCTTGAACTTTTAAATACAGCCATTGAACATACGGTTGATTTAATAACTGACAAACATCACCCTCTTGCTAAAGCGGCCAAGGACGCCGCCGCCGGGGCTGTTTGCGTTCTTGCCGTGATTTCGTGTACCATTGGTTTACTTATTTTTTTGCCAAGGCTGTAATGGCTGAAAATTCTTACATTTATTTTACATTTTTAGAAATGGGCGTGAAAAAAAGCGCGCGATTATGTAAAATATAAAGTGATAGCGGTACCATTATAGGTAAGAGAGGAATGTACAC |
| *lgtB* gene | ATGCAAAATCATGTCATTTCTTTAGCATCAGCAGCGGAAAGACGCGCTCATATTGCCGATACATTTGGCAGACATGGAATCCCGTTTCAATTTTTCGATGCGCTTATGCCGTCAGAACGCTTAGAACAGGCAATGGCGGAATTAGTTCCGGGCCTGTCAGCTCATCCGTATCTTAGCGGAGTGGAAAAAGCATGCTTTATGAGCCATGCGGTCTTATGGAAACAAGCTCTTGATGAAGGCCTGCCGTACATCACAGTTTTTGAAGATGATGTGCTGCTTGGCGAAGGAGCCGAAAAATTTCTGGCAGAAGATGCGTGGCTTCAGGAAAGATTTGATCCGGATACAGCATTTATCGTGCGCTTAGAAACAATGTTTATGCATGTCCTGACATCACCGAGCGGCGTTGCCGATTATTGTGGAAGAGCATTTCCGTTACTGGAATCTGAACATTGGGGCACAGCGGGATACATCATCTCAAGAAAAGCTATGAGATTTTTCCTGGATAGATTTGCTGCCCTTCCGCCGGAAGGCTTACATCCGGTTGATCTGATGATGTTTTCTGATTTCTTTGATCGCGAAGGAATGCCGGTGTGCCAACTGAATCCGGCTCTTTGTGCCCAGGAACTTCATTACGCCAAATTTCATGATCAAAACAGCGCACTGGGATCTCTTATCGAACATGATAGACTTCTGAACCGCAAACAACAGAGACGCGATAGCCCGGCGAACACATTTAAACATAGATTAATTCGCGCTCTGACAAAAATCTCTAGAGAACGCGAAAAAAGACGCCAAAGACGCGAACAGTTTATCGTTCCGTTTCAGTAA |
| Right homology arm | CTGATAAGAACTGCAAAAGCTGCGGATTATATGACAAATTGGTTCCATCGTACAGTGAAATATAAGATTTTTGTTGCTGAGCGGAATCATCGAGGGAAAAGTTCTCTTATCGGATGTCGAAAAAGAAGTCATTCAATCGTGAATACATAACATCATATTATTACATTCCTTTTCATCGATTGGAACAAGGGTTCTATACTTCAATGGAATACCGCAAAGTTGGAGTATTTAAACAACAGGGGAAAATCGATGGCCATTATGTAGGTGTCATGGCAATGGAAAAAATACTTGCAATGTAAACAGGCCTCTAAAGAGACCTGTTTTTTAATATCCAGATGATTGTCTAGTTTCAGCTTGGGCTCTATGACCACTCCCATAACTGAAATAGTCTAATATTTATACCAGAAGGGATTGTTTATATAAATAAAAGTTTATATAATGACGGGGGCGCTAAGGAAACGCGGGGAGCGACTCAGGTTTTCGTTTCAAAGTTTTGATCCTTAAGATATACGGAGCGCTACATATGGAAACGTAAAGGGGAATGTATAATCATTTGGCGTGTATATAACGATTTGCTTATATATTGATTTGGGAAGGAGGAGCTCATATGACGATTGATGTTGCGGCGATGACTCGTTGTTTGAAAACACTTAGTGATCAAACTAGGCTTATCATGATGAGATTATTTCTTGAACAAGAATATTGTGTCTGTCAATTGGTTGATATGTTCGAAATGAGCCAGCCCGCCATAAGCCAGCATTTGCGGAAATTAAAAAACGCAGGTTTTGTGAATGAGGACAGAAGAGGCCAATGGCGTTATTATTCAATAAATGGTTCCTGTCCTGAGTTTGATACATTGCAATTGATCTTACATCAAATTGATCAAGAGGATGAATTGTTAAACCATATCAAACAAAAGAAAACTCAAGCGTGTTGCCAGTAAGGAGGCATTAGATTGACATCAATGATTTTGGCAGTATTCATCTTTTTATTAACTCTAGTATTGGTCATATGGCAGCCGAAAAATTTATCTATAGGGTGG |

**Table S6 Genomic locus of 2 *lgtB* gene integration expression**

| **Name** | **Content (5’→3’)** |
| --- | --- |
| left homology arm | CATCCAGCGTAAAACGTTCACGGGAATAATCTAGGCCAAGACCTAATTTCGCCCACTGGCTGCGAATAAAGTCAGCATATTCTTCCTTCCACTTCCACGTTTCTTCGAGGAATTTTTCACGGCCTAAATCGTAGCGGGATTTGCCTTCTTCACGAAGTTTCGCCTCCACTTTAGCCTGTGTCGCGATGCCGGCGTGGTCCATGCCAGGAAGCCACAGCACATCATAGCCCTGCATGCGTTTCATTCTTGTTACAATGTCTTGCAGCGTTGTGTCCCATGCGTGGCCAAGGTGAAGTCTCCCTGTTACGTTTGGCGGCGGGATGACAACAGAGTATGGCTCTTTTGTCTGGTCACTGCCCGCTTCAAAAAATTTGCCTTTTAGCCAAAAATCATATCGGTCTTTTTCAACCGCTGCCGGATCATATTTCGTCGGCATTGTTTGTTCATTCGTTTCCATCATAACCCTCCAGCATGTTTAAAAATGGCTGAGAACGAAAAAAGAGCTCTTCATCCCTGTAAAAGGACGAAAAGCTCTTTCGCGGTACCACCTTTTTTCATGAACTCAATCAGTTCATGCACTTATAACGGATAACGGGTGTTTCCCGAATTTCTCTACTCTTCTTTCAAGAAATTATGCTCCCGGGCGACCTTCCAATCAGACAACATAAGAAGCCTTTCAGCAAACGGCTTCTCTCTCTGCATGCGCCTTTTTTGTACTCTTCCCGTTCTAAGCAGGTGTATTTATCAGCTCATTGTATACATTATTTATGTTCTTTATTATAGTAAAAGTGTTTTTGATTCGTCAATCATCTTTTCCAAAAACACCGCTTTCTATACAGATGGGCAGGCACCTATTGCCCAAGTTTACATATGATGCTTGTAAATGCGTAGAGGAGGATGCACCGTGAAGAAACGTTTCAGCTCTTATTCGCTGCCGCCATGGGTAAGGCAAATTCGGCTTGTATCCGCACAAGTGATTATTCCCATTACGATTTTTCAAGGAATCAGAACCATTTTCTTTCCGACAACCTTTGATGTTTTGCTGCTCGCAATCCTAATCTTTTTAGCTTGCGCCCTTCATTTAGAATGGATATAAACAAACAAGTCCGCGCCA |
| chloramphenicol resistance and P*_43_* promoter | GAGCGGATAACAATTTCACACAGGAAACAGCTATGACCATGATTACGAATTCGAGCTCGGTACCCGGGGATCCTCTAGAGATTGTACCGTTCGTATAGCATACATTATACGAAGTTATGCCATAGTGACTGGCGATGCTGTCGGAATGGACGACGGCAATAGTTACCCTTATTATCAAGATAAGAAAGAAAAGGATTTTTCGCTACGCTCAAATCCTTTAAAAAAACACAAAAGACCACATTTTTTAATGTGGTCTTTTATTCTTCAACTAAAGCACCCATTAGTTCAACAAACGAAAATTGGATAAAGTGGGATATTTTTAAAATATATATTTATGTTACAGTAATATTGACTTTTAAAAAAGGATTGATTCTAATGAAGAAAGCAGACAAGTAAGCCTCCTAAATTCACTTTAGATAAAAATTTAGGAGGCATATCAAATGAACTTTAATAAAATTGATTTAGACAATTGGAAGAGAAAAGAGATATTTAATCATTATTTGAACCAACAAACGACTTTTAGTATAACCACAGAAATTGATATTAGTGTTTTATACCGAAACATAAAACAAGAAGGATATAAATTTTACCCTGCATTTATTTTCTTAGTGACAAGGGTGATAAACTCAAATACAGCTTTTAGAACTGGTTACAATAGCGACGGAGAGTTAGGTTATTGGGATAAGTTAGAGCCACTTTATACAATTTTTGATGGTGTATCTAAAACATTCTCTGGTATTTGGACTCCTGTAAAGAATGACTTCAAAGAGTTTTATGATTTATACCTTTCTGATGTAGAGAAATATAATGGTTCGGGGAAATTGTTTCCCAAAACACCTATACCTGAAAATGCTTTTTCTCTTTCTATTATTCCATGGACTTCATTTACTGGGTTTAACTTAAATATCAATAATAATAGTAATTACCTTCTACCCATTATTACAGCAGGAAAATTCATTAATAAAGGTAATTCAATATATTTACCGCTATCTTTACAGGTACATCATTCTGTTTGTGATGGTTATCATGCAGGATTGTTTATGAACTCTATTCAGGAATTGTCAGATAGGCCTAATGACTGGCTTTTATAATATGAGATAATGCCGACTGTACTTTTTACAGTCGGTTTTCTAACGATACATTAATAGGTACGAAAAAGCAACTTTTTTTGCGCTTAAAACCAGTCATACCAATAAATAACTTCGTATAGCATACATTATACGAACGGTATGATAGGTGGTATGTTTTCGCTTGAACTTTTAAATACAGCCATTGAACATACGGTTGATTTAATAACTGACAAACATCACCCTCTTGCTAAAGCGGCCAAGGACGCCGCCGCCGGGGCTGTTTGCGTTCTTGCCGTGATTTCGTGTACCATTGGTTTACTTATTTTTTTGCCAAGGCTGTAATGGCTGAAAATTCTTACATTTATTTTACATTTTTAGAAATGGGCGTGAAAAAAAGCGCGCGATTATGTAAAATATAAAGTGATAGCGGTACCATTATAGGTAAGAGAGGAATGTACAC |
| *lgtB* gene | ATGCAAAATCATGTCATTTCTTTAGCATCAGCAGCGGAAAGACGCGCTCATATTGCCGATACATTTGGCAGACATGGAATCCCGTTTCAATTTTTCGATGCGCTTATGCCGTCAGAACGCTTAGAACAGGCAATGGCGGAATTAGTTCCGGGCCTGTCAGCTCATCCGTATCTTAGCGGAGTGGAAAAAGCATGCTTTATGAGCCATGCGGTCTTATGGAAACAAGCTCTTGATGAAGGCCTGCCGTACATCACAGTTTTTGAAGATGATGTGCTGCTTGGCGAAGGAGCCGAAAAATTTCTGGCAGAAGATGCGTGGCTTCAGGAAAGATTTGATCCGGATACAGCATTTATCGTGCGCTTAGAAACAATGTTTATGCATGTCCTGACATCACCGAGCGGCGTTGCCGATTATTGTGGAAGAGCATTTCCGTTACTGGAATCTGAACATTGGGGCACAGCGGGATACATCATCTCAAGAAAAGCTATGAGATTTTTCCTGGATAGATTTGCTGCCCTTCCGCCGGAAGGCTTACATCCGGTTGATCTGATGATGTTTTCTGATTTCTTTGATCGCGAAGGAATGCCGGTGTGCCAACTGAATCCGGCTCTTTGTGCCCAGGAACTTCATTACGCCAAATTTCATGATCAAAACAGCGCACTGGGATCTCTTATCGAACATGATAGACTTCTGAACCGCAAACAACAGAGACGCGATAGCCCGGCGAACACATTTAAACATAGATTAATTCGCGCTCTGACAAAAATCTCTAGAGAACGCGAAAAAAGACGCCAAAGACGCGAACAGTTTATCGTTCCGTTTCAGTAA |
| right homology arm | TTAAGACGTGGACTCGTTTTCAGCCTGAAATTTTTTCTCTTGTTCAATCTGATGAATTTTCATCACAAACGGCTCTATGTTTTTCATTTGCCAATAAGCCTTTAATAATTGTGTACATTCCTCTTTTTCATGCTGTCTTTTCCCAGTTTGATAACCGTTCAGCACCTTGTACATGGCTGCCGGATATGCCATATAGCTGAGAAAAAGGCTGACTTCCGCTTCACGCAAAGGGAATGATTTCGTATATCCATAAAACCATTCGACGCATTCCGGACAAGCTTTCGGAAAGCCTCTGAACATTTTTGTATAAAAGCCGAGCAGGTCGTTTTGCGGCGGACCGACAGATGCTCTTTCGAAGTTTGTAAAATATCCGGTCCCGGCATCATTATACAGAAAATGGTGTATAGACAGGCTGCCGTGGTTCATTACAACCCTTGAAACGTCCTTTTCCTTTGCGGACTCATACCAGTCCTCCAGCCGTTCCAGCGCAAAGTTCACCGCGGAAATGGTTTCAGAAAAATAGGTTATGGCCTGGAGTTCAAAGGGAGAAAGGTACCATTCCTTCTCCGCTCTATCCACAAACTGCTCATAGAAAATTTTATCTTGTTCCCACTTTTTTTTCGTTTGCCCGTAATAACGTTCAATCTCATCACGTCTAACCTTTAGTTCCTGCGCGGTTCTCTCGTGAAGACGGGCTGTTTCTCTAAATAAATAAGCATGTTTTTGATCCCGTTCTTCCTCTTGGTCAAATTGGAGCCAAGGCATTAAATAGTAGATATCTCCGTATTGTATGCCTGCTGAGAAAAACTCACCGCTATTTGTTCTGTAAACGGGAACGAATGAACGAAAGCCTTTTTCCTCAATCGCCACCATATGATCTGTAAAATGCTTGCCGCGACCTTCTTTCAATTTTTTCAGGGCAAATATGCCGTAGTCTGTATACACTTTCCAAACAAGAGGACTGACAGGCTCAATAAATTGAGCAGTCAGTCCGTATTCCTGAAGAACCGATTGGGTATCTTCCATTTCATTCACCACATTTTCCGATCACTTACGGTTTACGCATGGCTATTTTTATATTGAGGAATATAGAGAATCTGTCCTGCTTTTAATTCATCATCCAAGGCTAAAGAATTCATCCTGATCAGCTGCTGGGATG |

**Table S7** **Genomic locus of 3 *lgtB* gene integration expression**

| **Name** | **Content (5’→3’)** |
| --- | --- |
| left homology arm | GGCTTTCGTTGTCTTGTGTTCAAGAAATTTCCATGGTGTTTACTTTCCAGAATGATTCTTCGGAACAATGCTTTATGAATCTAACCCGCCTGACTTGGCGGATGATATAATCTTTATTTGCGTATAGAAAAGAATAAGACAAACCGCCCGGCGTACGCCGGACGGTTTTTTTATTGCAAAAAACACATCGTACTGAAACCTCCTTGTCTTCTTTCCAGTCTTATCTCTAGGATGAAATTGGAGGAAGAATAACGTGCATGTCATTACAACACAAGTACTTTTTATTTTTTGTTTTTTATTGCTGATTCACTCGATAGAAACCTTAGCCTATGCGACAAGGCTTTCCGGAGCTCGCGTTGGATTTATTGCGTCCGCGCTTTCTCTGTTTAATGTCATGGTCATCGTATCCAGAATGTCGAATATGGTGCAGCAGCCCTTTACTGGGCATTTAATTGATGATGCTGGAAAAAACGCACTGGCGATTGTAGGGGAGCAGTTCCGCTTTTTAATTTTCGGATCGACAGTCGGCACCATTTTGGGCATTATCCTGCTCCCGTCTTTTGTCGCTCTTTTTTCACGGGCGATTATTCACTTGGCGGGCGGCGGCGGCTCCGTTTTTCAAGTATTCCGAAAGGGATTCTCGAAACAAGGATTCAAAAATGCCCTTTCCTATTTGCGTCTGCCGTCCATTTCATATGTAAAAGGATTTCATATGCGCTTGATTCCGAAGCGTTTGTTTGTCATCAACATGCTGATCACATCGATTTATACGATTGGTGTGCTTTCGGCTTTATACGCAGGCCTTTTGGCGCCGGAGCGCAGCACGACAGCCGTCATGGCTTCGGGTTTGATCAACGGAATTGCAACGATGCTGCTGGCTATTTTTGTTGATCCTAAGGTATCCGTTCTTGCTGATGATGTGGCAAAAGGAAAACGAAGCTATATCTATTTAAAATGGACCTCTGTCACAATGGTCACATCAAGGGTGGCGGGCACACTCCTCGCCCAGCTCATGTTTATTCCCGGGGCCTACTATATCGCGTGGCTGACAAAGTGGTTTTAATAAGAAAGGCTGG |
| zeocin resistance and P*_43_* promoter | GAGCGGATAACAATTTCACACAGGAAACAGCTATGACCATGATTACGAATTCGAGCTCGGTACCCGGGGATCCTCTAGAGATACCGTTCGTATAGCATACATTATACGAAGTTATCTTGATATGGCTTTTTATATGTGTTACTCTACATACAGAAAGGAGGAACTAAACATGGCCAAGTTGACCAGTGCCGTTCCGGTGCTCACCGCGCGCGACGTCGCCGGAGCGGTCGAGTTCTGGACCGACCGGCTCGGGTTCTCCCGGGACTTCGTGGAGGACGACTTCGCCGGTGTGGTCCGGGACGACGTGACCCTGTTCATCAGCGCGGTCCAGGACCAGGTGGTGCCGGACAACACCCTGGCCTGGGTGTGGGTGCGCGGCCTGGACGAGCTGTACGCCGAGTGGTCGGAGGTCGTGTCCACGAACTTCCGGGACGCCTCCGGGCCGGCCATGACCGAGATCGGCGAGCAGCCGTGGGGGCGGGAGTTCGCCCTGCGCGACCCGGCCGGCAACTGCGTGCACTTCGTGGCCGAGGAGCAGGACTGAATAACTTCGTATAGCATACATTATACGAACGGTAAATCGTCGACTGATAGGTGGTATGTTTTCGCTTGAACTTTTAAATACAGCCATTGAACATACGGTTGATTTAATAACTGACAAACATCACCCTCTTGCTAAAGCGGCCAAGGACGCCGCCGCCGGGGCTGTTTGCGTTCTTGCCGTGATTTCGTGTACCATTGGTTTACTTATTTTTTTGCCAAGGCTGTAATGGCTGAAAATTCTTACATTTATTTTACATTTTTAGAAATGGGCGTGAAAAAAAGCGCGCGATTATGTAAAATATAAAGTGATAGCGGTACCATTATAGGTAAGAGAGGAATGTACAC |
| *lgtB* gene | ATGCAAAATCATGTCATTTCTTTAGCATCAGCAGCGGAAAGACGCGCTCATATTGCCGATACATTTGGCAGACATGGAATCCCGTTTCAATTTTTCGATGCGCTTATGCCGTCAGAACGCTTAGAACAGGCAATGGCGGAATTAGTTCCGGGCCTGTCAGCTCATCCGTATCTTAGCGGAGTGGAAAAAGCATGCTTTATGAGCCATGCGGTCTTATGGAAACAAGCTCTTGATGAAGGCCTGCCGTACATCACAGTTTTTGAAGATGATGTGCTGCTTGGCGAAGGAGCCGAAAAATTTCTGGCAGAAGATGCGTGGCTTCAGGAAAGATTTGATCCGGATACAGCATTTATCGTGCGCTTAGAAACAATGTTTATGCATGTCCTGACATCACCGAGCGGCGTTGCCGATTATTGTGGAAGAGCATTTCCGTTACTGGAATCTGAACATTGGGGCACAGCGGGATACATCATCTCAAGAAAAGCTATGAGATTTTTCCTGGATAGATTTGCTGCCCTTCCGCCGGAAGGCTTACATCCGGTTGATCTGATGATGTTTTCTGATTTCTTTGATCGCGAAGGAATGCCGGTGTGCCAACTGAATCCGGCTCTTTGTGCCCAGGAACTTCATTACGCCAAATTTCATGATCAAAACAGCGCACTGGGATCTCTTATCGAACATGATAGACTTCTGAACCGCAAACAACAGAGACGCGATAGCCCGGCGAACACATTTAAACATAGATTAATTCGCGCTCTGACAAAAATCTCTAGAGAACGCGAAAAAAGACGCCAAAGACGCGAACAGTTTATCGTTCCGTTTCAGTAA |
| right homology arm | ATCATAAAGATCCAGCCTTTTTGCGCTTCCTTATTTATAGTTCCTTAATCCGCGGTCGTACAAAAAATTTCCGAATGGGACGAAAGCGGCGATGAAGCCGGCAGCTGACCATTTCAGCGGCCATTTGACAGAGAAGGTTGCATAAGCCAAGACAAGCAAATACAAAATGAACAACCCGCCGTGAACCGAACCGACAATTGTCACCGCAAGCGGAAGGCCTGCCCAATATTTAAGCGGCATAGCGATGAACAACAGGATTAAGAGTGACATTCCTTCAATAAAACCCATCGTGCGAAGTCTTCCGATCGGCGTGTGCAGCATAAATCGCCCTCCTTGTGGACACGTTTTCATTTTATACTATAAACAATCCGGGGGGGCATATGACAGCTTTCAAAAAATGTTCGGAAAACATTCATTTTTACATGCCTTTTCTAGGGAACTGTACTTGTCATTTACAAAAATACCCGAGATAATGTGTACAAAATCAAAAAAGAAGGATGTTGAAATGAAACTTGACCAGATTGATCTGAATATCATTGAGGAGCTGAAGAAGGACAGCCGTTTGTCGATGAGGGAATTAGGCAGAAAAATTAAGCTGTCGCCTCCATCTGTAACAGAACGGGTAAGACAGCTTGAATCGTTTGGCATCATCAAGCAATACACGCTGGAGGTCGACCAGAAAAAACTGGGGCTTCCCGTTTCCTGCATTGTGGAAGCAACCGTTAAAAACGCGGATTATGAGCGGTTCAAAAGCTATATTCAAACATTGCCGAATATTGAATTTTGCTACCGGATTGCGGGTGCAGCCTGCTATATGCTGAAAATCAATGCCGAAAGCCTCGAAGCGGTAGAAGATTTCATTAACAAAACATCGCCCTACGCGCAAACCGTCACTCACGTCATTTTCTCAGAAATTGACACGAAAAACGGGCGCGGTTAGAGAGTGCCGCGCGAAGTCTGTTATAATAACAGGATGAGCGTGAAAGAAAGAGAAGTGATCAAGCATGTCAAAAACAGTTGTATTAGCTGAAAAACCTTCAGTCGGCCGGGATTTAGCCCGGGTACTGAAGTGCCATAAAAAAGGAAACGGTTATCTCGAAGGCGATCAATATATTGTGACTTGGGCTTTAG |

**Table S8 Genomic locus of 4 *lgtB* gene integration expression**

| **Name** | **Content (5’→3’)** |
| --- | --- |
| left homology arm | TTGCGGTACAAGTTGTGGCAAATTCCTACGGACTGACAGGCATTCCGATGATTTCAATCGTGCTGCCGTTTGGCGCCGCTAATTTTGTTCATTATATGATCGGTTTCTTGATCGCAGCCGTCTCTGCTTTTATAGCTACATTGTTTCTCGGGTTTAAAGAAGAGACAGAATAACTGGATTTATTCGATTTCATTCATAAAACGGGGGATGAAAGGACAAAAAAGCTATGAAAATTAAAAGAATCTTAAATCATAATGCTATCGTCGTAAAGGATCAAAATGAAGAGAAGATTCTCTTGGGTGCAGGAATTGCGTTTAACAAAAAGAAGAATGATATTGTCGATCCGTCAAAAATAGAAAAAACCTTTATCAGAAAAGATACACCTGACTATAAGCAGTTCGAAGAGATTTTAGAAACATTGCCTGAAGACCACATTCAGATTTCTGAGCAAATTATCTCTCATGCCGAAAAAGAGCTGAACATCAAAATCAACGAGCGCATTCATGTCGCTTTTTCAGACCATCTTTCTTTTGCAATTGAACGCCTGAGCAATGGGATGGTTATCAAAAATCCGCTGCTGAATGAAATCAAAGTCCTTTATCCAAAGGAGTTCCAGATCGGCTTATGGGCCAGAGCACTGATTAAAGATAAACTGGGGATTCACATTCCTGATGATGAAATCGGCAATATCGCCATGCATATCCACACAGCAAGAAACAATGCCGGCGATATGACACAAACGCTTGATATTACAACAATGATCCGTGATATTATCGAGATTATCGAAATTCAACTGTCTATTAATATCGTTGAAGATACCATCTCTTATGAAAGGCTCGTGACCCATCTCCGCTTTGCCATTCAGCATATCAAAGCAGGCGAATCCATTTACGAGCTGGACGCAGAAATGATTGACATCATTAAAGAGAAGTTTAAGGATGCCTTCCTGTGTGCCCTAAGCATCGGCACCTTTGTGAAGAAGGAATACGGCTTTGAGTTTCCTGAAAAAGAATTGTGCTACATCGCCATGCATATTCAGCGGTTCTACCAACGGTCAGTCGCACGCTGAGACAAACAAAAAACGCT |
| Spectinomycin resistance and P*_43_* promoter | GAGCGGATAACAATTTCACACAGGAAACAGCTATGACCATGATTACGAATTCGAGCTCGGTACCCGGGGATCCTCTAGAGATTGTACCGTTCGTATAGCATACATTATACGAAGTTATCGATTTTCGTTCGTGAATACATGTTATAATAACTATAACTAATAACGTAACGTGACTGGCAAGAGATATTTTTAAAACAATGAATAGGTTTACACTTACTTTAGTTTTATGGAAATGAAAGATCATATCATATATAATCTAGAATAAAATTAACTAAAATAATTATTATCTAGATAAAAAATTTAGAAGCCAATGAAATCTATAAATAAACTAAATTAAGTTTATTTAATTAACAACTATGGATATAAAATAGGTACTAATCAAAATAGTGAGGAGGATATATTTGAATACATACGAACAAGTTAATAAAGTGAAAAAAATACTTCGGAAACATTTAAAAAATAACCTTATTGGTACTTACATGTTTGGATCAGGAGTTGAGAGTGGACTAAAACCAAATAGTGATCTTGACTTTTTAGTCGTCGTATCTGAACCATTGACAGATCAAAGTAAAGAAATACTTATACAAAAAATTAGACCTATTTCAAAAAAAATAGGAGATAAAAGCAACTTACGATATATTGAATTAACAATTATTATTCAGCAAGAAATGGTACCGTGGAATCATCCTCCCAAACAAGAATTTATTTATGGAGAATGGTTACAAGAGCTTTATGAACAAGGATACATTCCTCAGAAGGAATTAAATTCAGATTTAACCATAATGCTTTACCAAGCAAAACGAAAAAATAAAAGAATATACGGAAATTATGACTTAGAGGAATTACTACCTGATATTCCATTTTCTGATGTGAGAAGAGCCATTATGGATTCGTCAGAGGAATTAATAGATAATTATCAGGATGATGAAACCAACTCTATATTAACTTTATGCCGTATGATTTTAACTATGGACACGGGTAAAATCATACCAAAAGATATTGCGGGAAATGCAGTGGCTGAATCTTCTCCATTAGAACATAGGGAGAGAATTTTGTTAGCAGTTCGTAGTTATCTTGGAGAGAATATTGAATGGACTAATGAAAATGTAAATTTAACTATAAACTATTTAAATAACAGATTAAAAAAATTATAAATAACTTCGTATAGCATACATTATACGAACGGTAGAATCGTCGACTGATAGGTGGTATGTTTTCGCTTGAACTTTTAAATACAGCCATTGAACATACGGTTGATTTAATAACTGACAAACATCACCCTCTTGCTAAAGCGGCCAAGGACGCCGCCGCCGGGGCTGTTTGCGTTCTTGCCGTGATTTCGTGTACCATTGGTTTACTTATTTTTTTGCCAAGGCTGTAATGGCTGAAAATTCTTACATTTATTTTACATTTTTAGAAATGGGCGTGAAAAAAAGCGCGCGATTATGTAAAATATAAAGTGATAGCGGTACCATTATAGGTAAGAGAGGAATGTACAC |
| *lgtB* gene | ATGCAAAATCATGTCATTTCTTTAGCATCAGCAGCGGAAAGACGCGCTCATATTGCCGATACATTTGGCAGACATGGAATCCCGTTTCAATTTTTCGATGCGCTTATGCCGTCAGAACGCTTAGAACAGGCAATGGCGGAATTAGTTCCGGGCCTGTCAGCTCATCCGTATCTTAGCGGAGTGGAAAAAGCATGCTTTATGAGCCATGCGGTCTTATGGAAACAAGCTCTTGATGAAGGCCTGCCGTACATCACAGTTTTTGAAGATGATGTGCTGCTTGGCGAAGGAGCCGAAAAATTTCTGGCAGAAGATGCGTGGCTTCAGGAAAGATTTGATCCGGATACAGCATTTATCGTGCGCTTAGAAACAATGTTTATGCATGTCCTGACATCACCGAGCGGCGTTGCCGATTATTGTGGAAGAGCATTTCCGTTACTGGAATCTGAACATTGGGGCACAGCGGGATACATCATCTCAAGAAAAGCTATGAGATTTTTCCTGGATAGATTTGCTGCCCTTCCGCCGGAAGGCTTACATCCGGTTGATCTGATGATGTTTTCTGATTTCTTTGATCGCGAAGGAATGCCGGTGTGCCAACTGAATCCGGCTCTTTGTGCCCAGGAACTTCATTACGCCAAATTTCATGATCAAAACAGCGCACTGGGATCTCTTATCGAACATGATAGACTTCTGAACCGCAAACAACAGAGACGCGATAGCCCGGCGAACACATTTAAACATAGATTAATTCGCGCTCTGACAAAAATCTCTAGAGAACGCGAAAAAAGACGCCAAAGACGCGAACAGTTTATCGTTCCGTTTCAGTAA |
| right homology arm | TTTGATCATCTCAAAAGCGTTTTTTTATCTGATTTATTGGTTGATCGCCGGATTCCCAATCGTATTCCACTTCGTATAGGACATGTAATGGAAATACTCATCGCGATACGGATGTTTTGTATTGGAGTTCCAAGGCTTTTCTCCGCCGCAGAAGTGCACGATTGCCGGGTTTTCTCTTGTTTCATTATATTGCTTCCGCCCTAACAGAGTCGATGGGGTTTTCAGCTTTAACATGATATAGGTTTGAGCGTTCCAGCGCGGATGAAGTTCATACCACTGATCATACAAGATCGCATTCAGTGCATCTTGGTCATGCAGCACAAGAAAGTCTTCGTCTGGGTGTTCATTGATAAAGTTGATGACTTTTTCTGTGATGTTTTGCTTTCTCCAAGACTCAAAATCAATAATCATGATACCTGAGTTAAAATACTTCCCTGTATCAGTGACGTTCATTTCTTTCAGGCGTTCATGCTGCCCCGCATCCTCAACAGCGGCTACGGTGTATGGCGCAATGTCTAAGTCCCATAGCTTAGAAATATCCTCTAGGACAAGCGCATCACAATCGATGTAAATCATTCGTTTGATGCTTTCATCCTTAATTAAGTCGGGAATCGAAATGCGGTAATACGCGGCTTTTGTAATATGGCTGCTCTCAACCGCATGTTCATACATGTTGGTATCCACTTCTAAAAACTCAATCGGCACTCCGAATTTCAAAGTGGTTTCTTCCAGTCTTTTTTTGTTATCAGGCTTAATTCCGCCATCAATGACGTATAATTTTACCTCTCTCTCCTGATCCATGTTAGTCAATAAAGAAACGAACATCCCACCTAAATGACGAGCATAATTATCATCTGCGCATGATACGATATGCATGATTTCATCTTTCCTCAATGGTATTCATCTCCCTTTTTGTTGTACAAGTTACATACCCTTTTTCAAAAAAATAAACACGTTCTCCGACATTTTTTTAAACAGAATGTGTGATACAACAAAAAAGGTGTCATTTTTTGAAGATGCGATAAAGCCAATAAAGAATGAAAAAGCTTGCGATTGTGATGGCTTCAGCCGAGACGA |

**Table S9** **Genomic locus of *pgcA* gene integration expression**

| **Name** | **Content (5’→3’)** |
| --- | --- |
| left homology arm | AGCTTCTGCTTTTCCATGTACTCCCCCCTCTGGCCGCTATGGTGACTTCATTATAGAAGGAGCAACATGATAGTTCATTGACAGATTAACCAAAAAAACGTTTATTTTTTAGCCACAGTATCCATCCAGCAACCAGATACACCCTACATGTCACAACTATTTTGATGTAAAGGTCAAAAGAAAACACGGTAAGGAAGAGAGCCAGCAAGAGCGAAAGAGAGGGTCAAAATGCCTTTCATATGAACAAGCTAAGCTGGAGAGGCGATGTTCGCGGAACGGATGAAAACAGAAATGCCGGCAGCCCGCAAAAACAGGACCTAAAGATATGGAAACAGGAGATTTCCGCCTCCTCCAAAGGGACTAATTTACAATTTTAATGAAAAATATACAAAATGACACTTCTATCCTTTATTTCAATTTTAGATAATAGATATGATCTGATAAAAGGAGGGCAAGTGATGACAAAAAAAGCATGGTTTCTGCCGCTCGTCTGTGTATTACTGATTTCCGGATGGCTTGCGCCAGCAGCTTCAGCAAGCGCGCAAACCACATTAAGCCTTAATGACCGATTGGCGTCTTCCCCTTCAGGAACGGGAAGCCTTCTCTCATTAGCCGCCCCCGCTGCACCCTATGCTGACACAGATACCTATTATGAAGGGGCTGAAGGTAAAACAGGAGACTCGCTAAAAAGCACCCTGCACCGTATTATCAGCGGACACACGATGCTGTCCTACAGCGAAGTGTGGAACGCGCTGAAAGAAACGGATGAAGACCCAAGAAATCCGAATAACGTCATCCTGCTCTATACGAATGAATCGCGTTCCAAAAACCTGAACGGCGGCAATGTCGGCGATTGGAACCGCGAGCATGTCTGGGCGAAATCCCATGGCGATTTTGGTACGAGCAAGGGACCTGGTACTGATATTCATCATTTGCGCCCGGCTGATGTTCAAGTTAACAGCGCCAGAGGAAATATGGATTTTGACAATGGCGGCACTGAATATGCGAAGGCACCCGGAAATTATTATGACGGCGATTCATGGGAGCCCCGCGATGATGTGAAAGGCGATGTTGCCCGCATGCTGTTTTACATGGCTGTCCGTTACGAGGGTGATGACGGCTATCCTGATCTTGAGCTTAATGATAAGACAGGCAACGGCTCAGCTCCTTATCATGGCAAACAATCTGTCCTGCTCGAATGGAATAAGCAGGATCCGGTTGACGACCGCGAGCGGAAAAGAAATGAAATCATTTATGAAAAATATCAGCACAACCGCAATCCATTTATCGACCACCCTGAATGGGCGGATGAGATTTGGCCGTAAGGAGCACCTTTTT |
| zeocin resistance and P*_43_* promoter | GAGCGGATAACAATTTCACACAGGAAACAGCTATGACCATGATTACGAATTCGAGCTCGGTACCCGGGGATCCTCTAGAGATACCGTTCGTATAGCATACATTATACGAAGTTATCTTGATATGGCTTTTTATATGTGTTACTCTACATACAGAAAGGAGGAACTAAACATGGCCAAGTTGACCAGTGCCGTTCCGGTGCTCACCGCGCGCGACGTCGCCGGAGCGGTCGAGTTCTGGACCGACCGGCTCGGGTTCTCCCGGGACTTCGTGGAGGACGACTTCGCCGGTGTGGTCCGGGACGACGTGACCCTGTTCATCAGCGCGGTCCAGGACCAGGTGGTGCCGGACAACACCCTGGCCTGGGTGTGGGTGCGCGGCCTGGACGAGCTGTACGCCGAGTGGTCGGAGGTCGTGTCCACGAACTTCCGGGACGCCTCCGGGCCGGCCATGACCGAGATCGGCGAGCAGCCGTGGGGGCGGGAGTTCGCCCTGCGCGACCCGGCCGGCAACTGCGTGCACTTCGTGGCCGAGGAGCAGGACTGAATAACTTCGTATAGCATACATTATACGAACGGTAAATCGTCGACTGATAGGTGGTATGTTTTCGCTTGAACTTTTAAATACAGCCATTGAACATACGGTTGATTTAATAACTGACAAACATCACCCTCTTGCTAAAGCGGCCAAGGACGCCGCCGCCGGGGCTGTTTGCGTTCTTGCCGTGATTTCGTGTACCATTGGTTTACTTATTTTTTTGCCAAGGCTGTAATGGCTGAAAATTCTTACATTTATTTTACATTTTTAGAAATGGGCGTGAAAAAAAGCGCGCGATTATGTAAAATATAAAGTGATAGCGGTACCATTATAGGTAAGAGAGGAATGTACAC |
| *pgcA* gene | ATGACTTGGAGAAAGAGCTATGAACGCTGGAAACAGACAGAACATTTAGATCTGGAATTAAAAGAGCGCCTTATTGAATTAGAGGGAGATGAACAGGCCCTTGAGGACTGTTTCTATAAAGACCTTGAATTCGGTACCGGCGGAATGCGCGGGGAAATCGGCGCCGGGACAAATCGGATGAATATTTACACTGTGCGCAAAGCATCGGCCGGGTTTGCGGCATACATCTCGAAGCAAGGTGAGGAAGCGAAAAAACGGGGCGTTGTCATTGCTTATGATTCCCGCCATAAGTCTCCGGAGTTCGCGATGGAAGCGGCAAAAACACTTGCGACACAAGGCATCCAAACATACGTGTTTGATGAGCTTCGCCCGACGCCAGAGCTGTCATTCGCTGTTAGACAGCTGAACGCTTATGGTGGAATTGTGGTAACGGCAAGCCATAACCCGCCTGAATATAACGGCTACAAAGTATACGGGGATGATGGCGGCCAGCTGCCTCCAAAGGAAGCGGACATCGTCATTGAGCAGGTAAACGCGATTGAAAATGAGCTGACGATCACAGTGGACGAAGAAAATAAGTTAAAAGAAAAAGGCTTAATCAAAATCATCGGTGAAGATATTGATAAAGTTTATACAGAAAAACTGACGTCCATTTCTGTACATCCTGAATTATCGGAAGAAGTAGATGTAAAGGTTGTTTTCACACCGCTGCATGGAACTGCAAATAAACCGGTCAGACGCGGTCTTGAAGCACTCGGCTACAAAAATGTAACGGTTGTCAAAGAACAGGAACTGCCGGATTCAAACTTCTCCACTGTTACATCGCCGAACCCGGAAGAGCATGCGGCATTCGAATATGCCATTAAGCTTGGGGAGGAGCAGAATGCAGATATTCTCATCGCGACAGATCCTGATGCTGACCGCCTCGGCATCGCGGTGAAAAACGATCAAGGCAAATATACAGTGCTGACAGGAAACCAAACCGGAGCGTTGCTGCTTCATTACCTGCTTTCTGAAAAGAAAAAACAAGGCATCCTGCCTGATAACGGTGTTGTTCTCAAAACGATCGTCACAAGCGAAATCGGCCGTGCTGTAGCTTCTTCATTCGGCCTTGATACGATTGATACGCTGACAGGCTTTAAGTTTATCGGTGAAAAGATTAAGGAATACGAAGCATCAGGCCAGTATACCTTCCAATTCGGTTATGAAGAGAGCTACGGTTATTTAATCGGGGATTTTGCCCGCGATAAGGACGCCATTCAGGCTGCGCTTTTGGCAGTTGAAGTTTGCGCGTTCTATAAAAAACAGGGAATGTCATTGTATGAGGCGCTCATCAATCTCTTTAACGAATATGGTTTTTATCGTGAAGGGCTGAAATCCCTGACGCTGAAAGGCAAACAAGGAGCAGAGCAAATTGAAGCGATTCTTGCTTCCTTCAGACAAAATCCGCCGCAGAAAATGGCGGGCAAACAGGTTGTCACAGCAGAAGATTACGCTGTAAGCAAACGGACGCTTCTGACTGAAAGCAAAGAAGAAGCCATCGACTTGCCAAAATCAAATGTATTGAAATACTTCCTGGAAGACGGGTCTTGGTTCTGTCTCCGTCCTTCTGGAACTGAGCCGAAGGTTAAATTTTATTTCGCCGTAAAAGGGTCATCTTTGGAAGACAGTGAAAAGCGACTTGCCGTCCTTTCTGAAGATGTAATGAAGACGGTGGATGAAATTGTTGAGTCAACAGCAAAATAA |
| right homology arm | CTAATACACAGCCTCTTCCGTTTCCGCATCGAAAAAGACGACGTGGTCCATTTTCACTGAGAGCTGGATTGAATCGCCTGCATCGATTCGCGTGTTTCCGTCCAGGCGGACCTTGAGCCGCTCGTCCCCAGCCATGACATGCACTATGAGCTCCGAGCCCAGATTTTCATTCACTTCCACGTTCGCTTGAAACACTGAATCAAACAACTGGTCATTTCCTGTCATTTGTGTGATGTGCTCCGGCCGTACACCGGCAATCATCTGTTCTCCGGCATATCCCTTTTCCTTCAGCCGCTTTGCTTTTTCTTCAGGAATATGAAGCCGGATCGAAGAATTTGTGAAAAACAGCTCGCCATGCTGCTGTTCTATGATGCCTTTCAAAAAGTTCATTCCGGGAGAGCCAATGAAGCCTGCGACAAACAAATTGGCCGGGTAATGATAAATGTCATGAGGTTTCGCCACTTGCTGAATTTCCCCTTCATTCATGACGACAATCCGATCTCCCATTGTCATTGCTTCGGTCTGGTCATGCGTGACATAAATGATCGTTGCCTCTAATCGCTGGTGCAGCTTGCTGATTTCTGTCCGCATCGTCACTCTTAGTTTTGCATCCAGATTAGAAAGCGGCTCGTCCATTAAAAAGACCTTCGGCTCCCTCACAATCGACCTGCCAAGCGCCACCCTTTGCCGCTGCCCGCCGGAGAGCGCCTTCGGTTTTCTCTTCAGCAAATGTTCAATTTCTAAAATTCTGGCTGCCGCATGAACCCGCTCGGCAATTTCCTGTTTCGCCATCTTTCTGAGCTTTAATCCAAACGCCATATTATCAAAAACCGTCATATGCGGATAAAGCGCATAGTTCTGGAATACCATCGCAATATCACGTTCTTTCGGCGGAAGATCATTGACCCGCTCCCCATCAATAAGAAGATTTCCTTCAGAAATGCTTTCTAATCCAGCCACCATCCGCAGCGTTGTTGATTTTCCGCATCCTGACGGCCCCACCAGCACCAAAAGCTCCTTATCCTTTACATCTAAATCAAAGTCCTTCACGGTTAATTGTGAGTGATATGATTTTTTGACGTGTTCAAATGTTAATGAAGCCATCAGAAAAGTCCCCCTATCTTTTGACCCGTTATTTCGTCACTGGAAACAACTTGGTCTTTCTGTTATTTTTAGAGTAATCGATTCCCATTTTTGCTATATTTCTAAAATAAAAAATGTAAATTTGTATTATAACGTCATAATACATTATAATATAAATAATAGGTTAAAAGGAGGCTACCCTTTGTTAAACAACGGAAGTTCTACACCTTTATACATTCAGCTAAAACAAATCATCACTGATGACATCAAAAAGGGCGTGTATTCCCCAACCGCCAAGCTGCCTACCGAAAACG |

**Table S10 Genomic locus of *gtaB* gene integration expression**

| **Name** | **Content (5’→3’)** |
| --- | --- |
| left homology arm | GGCAACGGTTCATCTTTTTCTCAGAACGGTAATCAATCATTCGGAAACCATTCACAGGCGCCTCAGCCGCCTAACGGACAAACCGGCGCTCTCAATGGAGGCGGAGGCACACCTCCCACAGGTGGCAACGGACCTGGAAATGGAGGGCCTGGCGGCGGAGGCGGAAAAAGCGTGAATATGTTCGGCACCGGTGATCCTGGACCGCTTCGTCTCTTCCAATCAGCACTTTCCGGCCAAATCAGCTGGATGCTTCCATTCTCATTGATCGGATTACTGGGCGCAATCATCAGCTGGTACCGTGATCGCCGCGGACATGCTGCTAAAATGAAAGAAACGCTATTCTGGGCGGCTTGGCTTGTTCCCGTGGCTGGTTTCTTCAGCATCGCAGGATTCTTCCACCAGTATTATTTGATTATGCTGGCACCGCCGATTGCCGCCCTTTCTGGTATCGGATGGTATACGATGCACCGCTTATACAAAAATAATAAAGACTGGTCCAGCTATCTTCTGCCGGCAGCCGTCTTGATCACAGCCGTGTTTCAGGTGTATATTCTGAGCGCTTATACAAGCCAAATCGGCAGTGTATGGATGTATGTCCTGGGGCTCTTAGGACTGGGCATCACCCTTGCACTTCTGATGCTTAAACGCAGCCATCCGTTCAGTAAGCTGCTGACCATCATCAGCTTGTGCGTTCTGCTTCTCACACCAGTTTACTGGTCAGCAACGCCGCTTCTATACGGTGGCAACAGCGTCCTTCCTGAATCCGGCCCGCAGCTCAAAGGCTCAGCAAACGGCGGGAACATGTTCTCTTCAGAGGTCGACAGCGGGCTCCTGTCCTATCTGAGAAAACACAATACCGGCGAAGAGTATTTATTTGCTACTTTGACAACCGTAACAGCTGCGCCATACATCATTTACGAAAATGAATCTGTTATGGCAATGGGCGGGTTTAACGGCACGGATCCCATTCTGACTGTGTCGGAACTGAAAAAACTGATCAAAGAAGGCAAAGTGAAATACTTCCTTCTGTCAGAAAACAACTCAGGCAACAGCGAACTTGTTTCCTGGATTAAGAAAAACGGAAAAGAAATAACATCTGACGAGTACAGCGGTTCCTCCAGCAGCACAAACAGCGTGCAGGGAATGCGAAGAGGACCTGGCGGAGAAAGCCAGCAAACTCTTTATCTCGTGGAATAA |
| spectinomycin resistance and P*_43_* promoter | GAGCGGATAACAATTTCACACAGGAAACAGCTATGACCATGATTACGAATTCGAGCTCGGTACCCGGGGATCCTCTAGAGATTGTACCGTTCGTATAGCATACATTATACGAAGTTATCGATTTTCGTTCGTGAATACATGTTATAATAACTATAACTAATAACGTAACGTGACTGGCAAGAGATATTTTTAAAACAATGAATAGGTTTACACTTACTTTAGTTTTATGGAAATGAAAGATCATATCATATATAATCTAGAATAAAATTAACTAAAATAATTATTATCTAGATAAAAAATTTAGAAGCCAATGAAATCTATAAATAAACTAAATTAAGTTTATTTAATTAACAACTATGGATATAAAATAGGTACTAATCAAAATAGTGAGGAGGATATATTTGAATACATACGAACAAGTTAATAAAGTGAAAAAAATACTTCGGAAACATTTAAAAAATAACCTTATTGGTACTTACATGTTTGGATCAGGAGTTGAGAGTGGACTAAAACCAAATAGTGATCTTGACTTTTTAGTCGTCGTATCTGAACCATTGACAGATCAAAGTAAAGAAATACTTATACAAAAAATTAGACCTATTTCAAAAAAAATAGGAGATAAAAGCAACTTACGATATATTGAATTAACAATTATTATTCAGCAAGAAATGGTACCGTGGAATCATCCTCCCAAACAAGAATTTATTTATGGAGAATGGTTACAAGAGCTTTATGAACAAGGATACATTCCTCAGAAGGAATTAAATTCAGATTTAACCATAATGCTTTACCAAGCAAAACGAAAAAATAAAAGAATATACGGAAATTATGACTTAGAGGAATTACTACCTGATATTCCATTTTCTGATGTGAGAAGAGCCATTATGGATTCGTCAGAGGAATTAATAGATAATTATCAGGATGATGAAACCAACTCTATATTAACTTTATGCCGTATGATTTTAACTATGGACACGGGTAAAATCATACCAAAAGATATTGCGGGAAATGCAGTGGCTGAATCTTCTCCATTAGAACATAGGGAGAGAATTTTGTTAGCAGTTCGTAGTTATCTTGGAGAGAATATTGAATGGACTAATGAAAATGTAAATTTAACTATAAACTATTTAAATAACAGATTAAAAAAATTATAAATAACTTCGTATAGCATACATTATACGAACGGTAGAATCGTCGACTGATAGGTGGTATGTTTTCGCTTGAACTTTTAAATACAGCCATTGAACATACGGTTGATTTAATAACTGACAAACATCACCCTCTTGCTAAAGCGGCCAAGGACGCCGCCGCCGGGGCTGTTTGCGTTCTTGCCGTGATTTCGTGTACCATTGGTTTACTTATTTTTTTGCCAAGGCTGTAATGGCTGAAAATTCTTACATTTATTTTACATTTTTAGAAATGGGCGTGAAAAAAAGCGCGCGATTATGTAAAATATAAAGTGATAGCGGTACCATTATAGGTAAGAGAGGAATGTACAC |
| *gtaB* gene | ATGAAAAAAGTACGTAAAGCCATAATTCCAGCAGCAGGCTTAGGAACACGTTTTCTTCCGGCTACGAAAGCAATGCCGAAAGAAATGCTTCCTATCGTTGATAAACCTACCATTCAATACATAATTGAAGAAGCTGTTGAAGCCGGTATTGAAGATATTATTATCGTAACAGGAAAAAGCAAGCGTGCGATTGAGGATCATTTTGATTACTCTCCTGAGCTTGAAAGAAACCTAGAAGAAAAAGGAAAAACTGAGCTGCTTGAAAAAGTGAAAAAGGCTTCTAACCTGGCTGACATTCACTATATCCGCCAAAAAGAACCTAAAGGTCTCGGACATGCTGTCTGGTGCGCACGCAACTTTATCGGCGATGAGCCGTTTGCGGTACTGCTTGGTGACGATATTGTTCAGGCTGAAACTCCAGGGTTGCGCCAATTAATGGATGAATATGAAAAAACACTTTCTTCTATTATCGGTGTTCAGCAGGTGCCCGAAGAAGAAACACACCGCTACGGCATTATTGACCCGCTGACAAGTGAAGGCCGCCGTTATCAGGTGAAAAACTTCGTTGAAAAACCGCCTAAAGGCACAGCACCTTCTAATCTTGCCATCTTAGGCCGTTACGTATTCACGCCTGAGATCTTCATGTATTTAGAAGAGCAGCAGGTTGGCGCCGGCGGAGAAATTCAGCTCACAGACGCCATTCAAAAGCTGAATGAAATTCAAAGAGTGTTTGCTTACGATTTTGAAGGCAAGCGTTATGATGTTGGTGAAAAGCTCGGCTTTATCACAACAACTCTTGAATTTGCGATGCAGGATAAAGAGCTTCGCGATCAGCTCGTTCCATTTATGGAAGGTTTACTAAACAAAGAAGAAATCTAA |
| right homology arm | TTAAGCTTCTTCTTTTACATGTACCTTTAACACAGCCTGCACTTCAGGATGCAGCTTCACAGGCACGTTTGTATATCCTAATGCACGAATGCCGTCCGGCAGTTCAAGCTTCCGCTTATCTACTTTAATGTTATGGTCTTTTTGGAGCTGTTCAGTGATTTGCTTGCTTGTCACTGAACCAAATAAACGGCCGCCCTCACCTGATTTTGCGCTAAGCTCAACAGTCAGTTTTTCTAATGTCTCTTTCAAGCTCTTAGCCTGCTCAAGCTCAGCAATGGCTTCTTTTTTCTCTTTCTGCTTTTGTCCGTTTAACGCGCTGATGTTTGACGCGTTCGCTTCAACCGCGAGGCCTTTTTTAATTAGAAAGTTATGCGCATAACCGTCTGCTACGTTTTTGACTTCGCCTTTTTTTCCTTTTCCTTTTACATCTTGTAAGAAAATAACCTTCATCTCTGTACGCCTCCCTCAAAATACTCATCTATGGCGTGCTTCAGCCGTTCCAGCGCTTCAGAAACTGAAATGCCGGACAACTGAGTCGCCGCATTTGTTAAATGCCCTCCGCCTTCCAGGGCCTCCATGATGATCTGAACATTTACCTCGCCAAGCGACCGAGCACTGATACAAACCGTTTGTTCATCCCGTCTCGCCACCGCAAATGAAGCCTCAACCTCACTCATCGATAGCAGCGAATCGGCAGCCTGCGCAATCAGCACTTGATCGAAGTATTCCTCTTCATTTTCAGGAAGAGAAGCAATCGCAATGTTATCTTTATAGAGGACCGTATGCTGGATCAGCTTCGCCCGTTTAATATAGGAATCAACGGTTTCTTTCAGAAACTTCTGCACCAGCACCGTGTCGGCGCCTTTTGCCCTTAAATAAGAAGCCGCATCGAAAGTCCGCGAGCCCGTGCGGAGAGAAAAGCTCTTTGTATCCACTATTATACCAGCTAATAGGGCTGTTGCTTCAATCATATTGATTTTTAAGCGCTTCGGCTGATATTCAAGCAGCTCTGTCACCAATTCCGCTGTGGAAGAAGCGTATGGTTCCATATAAACGAGCAGCGGATCTCTGATAAACTCCTCACCTCTTCGGTGATGG |

**Table S11 Genomic locus of *galE* gene integration expression**

| **Name** | **Content (5’→3’)** |
| --- | --- |
| left homology arm | GTGAAGCGGCGTATTATTAAAGGAAAGGCTGTATACTTTTTGCAGCTTTAAAAAGACTGAATATTTAAACAATTATGTGAAAGGTGTGCTGATTAGATTGAATAAGCTTATTGAACGAGAAAAAACTGTATATTATAAGGAAAAGCCCGACCCGTCTTCCTTGGGGTTTGGACAATATTTTACAGATTATATGTTTGTGATGGACTACGAAGAGGGGATTGGATGGCATCATCCGAGAATTGCGCCGTACGCACCGCTTACGCTTGATCCGTCTTCATCTGTTTTTCATTACGGCCAGGCTGTTTTTGAAGGATTAAAAGCATACAGAACAGACGACGGCAGGGTGCTGCTGTTCCGTCCGGATCAAAATATCAAACGGCTGAACAGATCGTGTGAGCGCATGAGCATGCCCCCTTTAGACGAAGAGCTGGTGCTTGAGGCATTGACGCAATTAGTTGAGCTGGAGAAAGATTGGGTTCCAAAGGAAAAAGGAACGTCACTGTATATTCGTCCTTTTGTCATTGCCACAGAACCGAGTCTCGGTGTGAAGGCATCCAGGAGCTATACATTTATGATCGTGCTTTCGCCTGTCGGCTCCTATTATGGCGACGATCAGCTGAAGCCGGTTAGAATCTATGTCGAAGATGAGTATGTGAGGGCGGTCAACGGAGGAGTCGGGTTTGCAAAAACGGCTGGAAACTATGCCGCCAGTCTTCAGGCACAGCGGAAAGCGAATGAACTGGGCTATGACCAGGTACTGTGGCTGGACGCCATCGAAAAGAAATATGTGGAAGAAGTAGGGAGCATGAACATCTTTTTCGTCATAAACGGGGAAGCTGTCACACCTGCTTTAAGCGGAAGCATTTTAAGCGGGGTTACACGTGCGTCTGCGATTGAATTGATTCGAAGCTGGGGCATTCCGGTTCGTGAAGAGAGAATATCGATTGATGAGGTGTATGCGGCCTCTGCACGCGGAGAATTGACAGAGGTCTTTGGCACAGGCACGGCAGCAGTCGTTACGCCTGTCGGTGAACTCAACATCCATGGAAAAACGGTGATTGTAGGCGACGGGCAAATCGGGGACCTCTCGAAAAAGCTGTATGAAACGATAACAGATATTCAGCTTGGCAAGGTAAAAGGCCCGTTTAACTGGACAGTGGAAGTGTGA |
| chloramphenicol resistance and P*_43_* promoter | GAGCGGATAACAATTTCACACAGGAAACAGCTATGACCATGATTACGAATTCGAGCTCGGTACCCGGGGATCCTCTAGAGATTGTACCGTTCGTATAGCATACATTATACGAAGTTATGCCATAGTGACTGGCGATGCTGTCGGAATGGACGACGGCAATAGTTACCCTTATTATCAAGATAAGAAAGAAAAGGATTTTTCGCTACGCTCAAATCCTTTAAAAAAACACAAAAGACCACATTTTTTAATGTGGTCTTTTATTCTTCAACTAAAGCACCCATTAGTTCAACAAACGAAAATTGGATAAAGTGGGATATTTTTAAAATATATATTTATGTTACAGTAATATTGACTTTTAAAAAAGGATTGATTCTAATGAAGAAAGCAGACAAGTAAGCCTCCTAAATTCACTTTAGATAAAAATTTAGGAGGCATATCAAATGAACTTTAATAAAATTGATTTAGACAATTGGAAGAGAAAAGAGATATTTAATCATTATTTGAACCAACAAACGACTTTTAGTATAACCACAGAAATTGATATTAGTGTTTTATACCGAAACATAAAACAAGAAGGATATAAATTTTACCCTGCATTTATTTTCTTAGTGACAAGGGTGATAAACTCAAATACAGCTTTTAGAACTGGTTACAATAGCGACGGAGAGTTAGGTTATTGGGATAAGTTAGAGCCACTTTATACAATTTTTGATGGTGTATCTAAAACATTCTCTGGTATTTGGACTCCTGTAAAGAATGACTTCAAAGAGTTTTATGATTTATACCTTTCTGATGTAGAGAAATATAATGGTTCGGGGAAATTGTTTCCCAAAACACCTATACCTGAAAATGCTTTTTCTCTTTCTATTATTCCATGGACTTCATTTACTGGGTTTAACTTAAATATCAATAATAATAGTAATTACCTTCTACCCATTATTACAGCAGGAAAATTCATTAATAAAGGTAATTCAATATATTTACCGCTATCTTTACAGGTACATCATTCTGTTTGTGATGGTTATCATGCAGGATTGTTTATGAACTCTATTCAGGAATTGTCAGATAGGCCTAATGACTGGCTTTTATAATATGAGATAATGCCGACTGTACTTTTTACAGTCGGTTTTCTAACGATACATTAATAGGTACGAAAAAGCAACTTTTTTTGCGCTTAAAACCAGTCATACCAATAAATAACTTCGTATAGCATACATTATACGAACGGTATGATAGGTGGTATGTTTTCGCTTGAACTTTTAAATACAGCCATTGAACATACGGTTGATTTAATAACTGACAAACATCACCCTCTTGCTAAAGCGGCCAAGGACGCCGCCGCCGGGGCTGTTTGCGTTCTTGCCGTGATTTCGTGTACCATTGGTTTACTTATTTTTTTGCCAAGGCTGTAATGGCTGAAAATTCTTACATTTATTTTACATTTTTAGAAATGGGCGTGAAAAAAAGCGCGCGATTATGTAAAATATAAAGTGATAGCGGTACCATTATAGGTAAGAGAGGAATGTACAC |
| *galE* gene | ATGGCAATACTTGTTACTGGCGGTGCCGGTTACATTGGCAGCCACACATGTGTTGAACTATTGAACAGCGGCTACGAGATTGTTGTTCTTGATAATCTGTCCAACAGTTCAGCTGAAGCGCTGAACCGTGTCAAGGAGATTACAGGAAAAGATTTAACGTTCTACGAAGCGGATTTATTGGACCGGGAAGCGGTAGATTCCGTTTTTGCTGAAAATGAAATCGAAGCTGTGATTCATTTTGCAGGGTTAAAAGCAGTCGGCGAATCTGTGGCGATTCCCCTCAAATATTATCATAACAATTTGACAGGAACGTTTATTTTATGCGAGGCCATGGAGAAATACGGCGTCAAGAAAATCGTATTCAGTTCATCTGCGACAGTATACGGCGTTCCGGAAACATCGCCGATTACGGAAGACTTTCCATTAGGCGCGACAAATCCTTATGGGCAGACGAAGCTCATGCTTGAACAAATATTGCGTGATTTGCATACAGCCGACAATGAGTGGAGCGTTGCGCTGCTTCGTTACTTTAACCCGTTCGGCGCGCATCCAAGCGGACGGATCGGTGAAGACCCGAACGGAATCCCAAATAACCTTATGCCGTATGTGGCACAGGTAGCAGTCGGGAAGCTCGAGCAATTAAGCGTATTCGGAAATGACTATCCGACAAAAGACGGGACAGGCGTACGCGATTATATTCACGTCGTTGATCTCGCAGAAGGCCACGTCAAGGCGCTGGAAAAAGTATTGAACTCTACAGGAGCCGATGCATACAACCTTGGAACAGGCACAGGCTACAGCGTGCTGGAAATGGTCAAAGCCTTTGAAAAAGTGTCAGGGAAAGAGGTTCCATACCGTTTTGCGGACCGCCGTCCGGGAGACATCGCCACATGCTTTGCAGATCCTGCGAAAGCCAAGCGAGAACTAGGCTGGGAAGCGAAACGCGGCCTTGAGGAAATGTGTGCTGATTCCTGGAGATGGCAGTCTTCTAATGTGAATGGGTATAAGAGTGCGGAATAA |
| right homology arm | AAATCGAAAAAGAACCTGCCCGGAGGCAGGTTCTTTTTATTTTGAATGAGTCATATCAGCAGCCGGCTGGCTTTTGCGGTTTTTTACATAATATATTGCATAGCAAATGGCCATAAATGGAACGCCGCAATAGAGTGCGATTCTTTGATTCGGATCAAAGGCAATGCCAACGACGGAAGCCAGGCATAACAGGAACGCAGCAATCGGCACGAACGGATACAAAGGGGTTCTGTATTTCAAATCAGTAACCTTGTTTCCCGCTTCGATATAACGTTTTCTGAACATGAATTGAGAAGCAGCGATTCCCATCCACACGACAACGACGGCAAAACCGGAAATCGACACGAGCACAACGTATACAGTGTCTGGCGCAAATACGCTGGACAGCAACGACAGAATGCCGCCGATCATGCTGAACACCAGCGCGTTAAAAGGGGTTCCTTTTGACGTCAGCTTTGCAAATGTCGGATGAAGCGTTTTTTCTTTTGATAAAGACCACAGCATCCGGGAAGAAGCGTAGAGTCCGGAATTAGCCGCTGACAGGATCGCTGTCAGGATAACAAAGTTCATGATATCGGCTGCGTATGGTACACCGACTCTGTCAAAGACAGCGACAAACGGGCTTTTGATGACACCTGCATCTTGAATCGGAATCAGCCCTGACAGCACAAAAATTGTTCCGACAAAGAAGAGCGAGAGACGCCAGACCGTTGTTTTTATCGCTTTTGGTATCGTCTTGTCAGGATCAACGCTTTCACCGGCTGCGATTCCGATTAGTTCCGTTCCCGAAAAGGCAAAATTGACGGACAGCATCGTCATCAAAATCGGGACAAATCCATTGGGAAAAAGCCCGCCTTCCGCCGTGAAGTTGGACAGCATCGGAGCGGCTTCTCCGCCTTTGATCGGGATGATGCCGAACATGGCAGAGCCGCCTAAAAGAATAAATAATACAATGGCTAATACTTTGATGCTGGAAAACCAAAATTCTGATTCAGCGAAAAACTTGACCGAAAATGCGTTAAGCAAGAAGATGAACAGCGCAAAGACGGCACTCCACATCCAAACCGATGTATGGGGGAACCAGCGCTGCATTAACAGGCCGGCTGCTGTGAATTCTGATCCCAATGCCACGGTCCATGTCAGCCAATACAGCCAAGCGACCGTAAATCCGG |

**Table S12 Genomic locus of *pgi* gene integration expression**

| **Name** | **Content (5’→3’)** |
| --- | --- |
| left homology arm | GAGGGTACAAAGATGTGAACTATACGCTTGCGGCCGCTTTTGTCTCTTACTGGGTCATTGGCCTTCCGGTCGGTTATATGGTCGGTACGTTTACAAGCCTCGGTGCATTCGGCTATTGGATTGGATTAATAGCGGGACTCGCCGCCGGAGCAGTTGGGCTGTTTTTCAGGCTGGCAAAACTGCAAAAACGTTATTCGCAAAAACAACACATTTAAAAGAGGTGATCAATCATGGCAAAAAACAAAAAATTATTCGAGTATCTTTCCCAGCATGCGGAAACCATCAGTTCAACATGGTATGAAACAATCGAGGAGACTGATCCCAATTCCATTTATGCCTCAACAGACCCTGTTGTCATTCACAATTTAAAGAGCCAGAATCTTGCCTTTAACTATAAAATAAACCGTATTTTTATAGATGATGAGGACGTATACTTGCCTATACTGAAAGAATGGGCCTTTGAAGTCACACAGGATCAAGAGCATTTAAAAACTCCTATCCACTATATTATTCGGGAGTTTGTCAGAGTGAGAGATTTATATGTTTCTTATGTGAAAGAATTTGTCCATCTCAATCAGAATACTGTGAAAAGCGAAGAAGCCGAAGACCTGTATCACGCCCTTATAAAAGCGTTTGATCTTGTGATTCATATTTTTATAGAAGAAATGTACAAAAACACAAGCCTTCAGCTTCAGGCCCAAAAAGATATGATTACTGAATTGAGCGCTCCGGTCATCGTGCTGTTTCACAGCGTCGGACTGCTGCCTTTAATCGGAGATATTGATACAGTCCGCGCCAAGCTGATTATGGAAAACACACTGCATCAATGTGCGAAAAAAAAGGTGACACAACTGTATATTGATTTGTCAGGAGTAGCTGTGATTGATACGATGGTTGCCCATCAGCTGTTCAGTCTGATTGAGGCGCTTCGTTTAATCGGTGTTTCTTCAACTTTGTCTGGAATCCGTCCGGAAATCGCGCAAACAGCGGTTCAGCTAGGTTTGTCTTTCGAAGGTATTTCCTTGAGATCCACTCTCGCTTCAGCTATTGCATCGGACTTAAAATTAAAAAAGGTATAAGGCTTAAGGCCTT |
| zeocin resistance and P*_43_* promoter | GAGCGGATAACAATTTCACACAGGAAACAGCTATGACCATGATTACGAATTCGAGCTCGGTACCCGGGGATCCTCTAGAGATACCGTTCGTATAGCATACATTATACGAAGTTATCTTGATATGGCTTTTTATATGTGTTACTCTACATACAGAAAGGAGGAACTAAACATGGCCAAGTTGACCAGTGCCGTTCCGGTGCTCACCGCGCGCGACGTCGCCGGAGCGGTCGAGTTCTGGACCGACCGGCTCGGGTTCTCCCGGGACTTCGTGGAGGACGACTTCGCCGGTGTGGTCCGGGACGACGTGACCCTGTTCATCAGCGCGGTCCAGGACCAGGTGGTGCCGGACAACACCCTGGCCTGGGTGTGGGTGCGCGGCCTGGACGAGCTGTACGCCGAGTGGTCGGAGGTCGTGTCCACGAACTTCCGGGACGCCTCCGGGCCGGCCATGACCGAGATCGGCGAGCAGCCGTGGGGGCGGGAGTTCGCCCTGCGCGACCCGGCCGGCAACTGCGTGCACTTCGTGGCCGAGGAGCAGGACTGAATAACTTCGTATAGCATACATTATACGAACGGTAAATCGTCGACTGATAGGTGGTATGTTTTCGCTTGAACTTTTAAATACAGCCATTGAACATACGGTTGATTTAATAACTGACAAACATCACCCTCTTGCTAAAGCGGCCAAGGACGCCGCCGCCGGGGCTGTTTGCGTTCTTGCCGTGATTTCGTGTACCATTGGTTTACTTATTTTTTTGCCAAGGCTGTAATGGCTGAAAATTCTTACATTTATTTTACATTTTTAGAAATGGGCGTGAAAAAAAGCGCGCGATTATGTAAAATATAAAGTGATAGCGGTACCATTATAGGTAAGAGAGGAATGTACAC |
| *pgi* gene | ATGACGCATGTACGCTTTGACTACTCAAAAGCGTTGACTTTCTTCAACGAACATGAACTTACATACCTGCGGGACTTTGTAAAAACAGCACACCATAATATCCATGAGAAAACAGGCGCGGGCAGCGATTTTCTAGGCTGGGTGGACCTCCCTGAACATTATGATAAAGAAGAATTCGCGCGCATCAAAAAAAGCGCGGAAAAAATCAAATCTGACTCTGATGTCTTGCTTGTTGTCGGCATCGGCGGTTCTTATCTTGGAGCGCGGGCAGCGATTGAAGCGCTGAATCACGCGTTTTATAACACTTTGCCAAAAGCAAAACGCGGCAATCCGCAAGTCATTTTTATCGGGAACAACATCAGTTCATCTTATATGAGAGACGTCATGGATCTTCTTGAAGATGTTGACTTCTCTATTAATGTGATTTCTAAATCAGGTACGACAACTGAACCTGCAATCGCTTTCCGTATTTTCCGCAAGCTTCTTGAAGAGAAATACGGTAAAGAAGAAGCGAAAGCGCGGATTTATGCAACAACTGATAAAGAGCGCGGCGCATTAAAAACGCTTTCTAACGAAGAAGGCTTTGAATCATTCGTAATTCCTGACGATGTCGGCGGCCGTTATTCAGTTTTAACAGCTGTAGGTCTCTTGCCGATTGCTGTCAGCGGCGTCAACATTGACGACATGATGAAAGGCGCCCTGGATGCGAGCAAAGATTTTGCAACATCTGAACTGGAAGATAACCCAGCATACCAATATGCGGTTGTTCGCAATGTCCTTTATAATAAGGGCAAAACAATTGAAATGCTCATCAACTACGAACCGGCGCTTCAATACTTTGCGGAATGGTGGAAGCAGCTGTTCGGAGAAAGCGAAGGGAAAGATGAGAAGGGCATTTATCCTTCTTCAGCGAACTATTCAACAGACCTTCATTCTTTAGGCCAGTATGTACAAGAAGGCCGCAGAGATTTATTCGAAACGGTCCTGAACGTAGAGAAGCCTAAACATGAACTGACAATTGAGGAAGCGGATAACGATCTTGACGGCTTGAACTATTTAGCCGGTAAAACTGTTGATTTCGTTAACAAAAAAGCATTCCAAGGTACAATGCTTGCCCATACAGACGGAAATGTTCCGAACTTAATCGTTAACATTCCTGAGCTGAATGCATATACTTTTGGATACCTTGTATATTTCTTCGAAAAAGCCTGCGCGATGAGCGGTTACCTCCTTGGCGTCAATCCGTTTGACCAGCCTGGTGTAGAAGCGTATAAAGTCAATATGTTTGCGTTACTCGGCAAACCTGGCTTTGAAGAGAAAAAAGCAGAGCTTGAAAAACGTCTGGAAGATTAA |
| right homology arm | ATACCTTTTTGATTAATCATTCCATTGATACGTCCAATATATTTCTTCTTCAAACCATGTTGCCATAACAGGTTCATTATTTTTCAATTTCTCTTCAAGCTCTCTGAGCATGCCTTCTGTTTGTGTGCGGTGAGCTCTTAACGCATTCATTTTAATATCAGCTACTTCTTTGATATCAAGTACGACATCAGCTTCGCCGAGAACCTCTTCTCTGTTGCGGGTAATGGCCATGCAGATTGTACGCGGACGGTCTTCTTTCTTTTTGCGGTATAATGCACGGATAACCGCTTCCCCGCATGCATCATGGTCAGGGTGCACACCGTGTCCGGGATAAAATGTGACAATTAAACTTGGTTTTACATCATCTATGATCTCTTCCATGATATCAGCTAAATACTCATCGTCTTCAAATTCGAGCGTTTTGTCACGGAGTCCAAGCATCCGAAGGTCATTGATATCCATTTCTTTGCAAGCGTTGATGAGCTCTTGTTTTCTTAGCAGCGGCAACGTTTCCCGATTCGCGAAAAATGGATCGCCCATATTTCTGCCCATTTCACCTAAAGTGGCGCAGGCATACGTAACGGGAATGTCTTTTTTTCTGTTTAAGGCAATGAGGCCTGCCACTCCATATGATTCATCATCAGGGTGAGGCAGGATTACTAGTACATGTTCTTTCATTATTATTCACCCTTTTCTTTAAACTGTGAACGGTTTTTCGCTGATTTGCAGAGAAATAGCCAGTTTACCGCCCGGCAGGTGTCCCGCCATTAATAAACGGTTTTCTTCATCTACCGTGTATTCAGTCAAGCCTTCAGCATAAATCCAGCCTTCTTCTGTTTTCAAGCCAACGCGGTAAGGCCCGTTGCCTTTGATTTTTGCCTGATGATAAGTGACTTTTGCATTGCGAATATACGCAACAACCGTCATGTTCTTTTCATTTAAGTGCGCTGAATAAGAACCTGTGGTGGTTTCTAAATGGATATATACAGGGCGGTCCGCATATCGTTCTAAAGATGCTTGCACATCTTCTTTTATGATCGCTTTCATG |

**Table S13 Genomic locus of *glmM* gene integration expression**

| **Name** | **Content (5’→3’)** |
| --- | --- |
| left homology arm | GGACAGGTTTTCTGTTTGAATGAAGCCGTCTATAATGAGCGGCATTTCGAGCTGCCGATGCTCTCCGCTGTTTTCTTTCTTAACAATAGTCAGTTTGCCCGACGGCTCTAAAATAGCAAATGATACGTCAGCTACTCTGTCGATGCTATTTTCTCTGAGCTGAACCATTAAATCATCAAAATTATAACGCTGTGATTTCATGGCTTCTTCGTCAATTTTTCCATATTTGATAATTATGGTCGGCTTACCGTCAAGAAGCTGGCGGACTTTTCGGTTTTTTAAAGAAAAGTATGCCAAAGTCACTTGAATGATCATTAATACAAGCATTGGCAAAATGGTATGAAATAGATGATCGTCTACGTTTTCTATGGCTAAAACGGCAATTTCAGCCATCATGATAAAGACAACGAGATCTAAAATACTAAGCTCTCCGATCTCCCGTTTGCCCATGAAACGAAAAATGACCAATATCACAAAATACAATACGACTGTGCGAAACGCAATAGTGAGAAGCTCTTCCATTATGCACCTCCAACTCATTATAGGTTGCAACAAAATGATCAATTTATGTAAGAAAAACCGATTGCATTTCACAAAGCTTTTACGTCTAATTCATGGGATAAGGGAATACATTTTTACAAAGACGAGCCATCAGCATGTCTGACGGTTTTGAACAAAAAGAACAGAAGCCTTTTAATACAGGCAGAGAGGAGACAATCATGGAGGAAACGAAACAAGTTGGAAAAGGCATTCTTTACGGCTTAATCGCCATATTTTCAGCCATGCTCCTGACAAGTTTAGCGGTTTCACTTTTATTGACCGCAACCTCGCTGGAAGAATCATCATTTAACTGGCTGATCACCGCTATCTCATTTCTCTCTTTATTTATCGGCGGGTTCATTTCAGGCGGGAAAGGCAAGGAAAGAGGCTGGATGATTGGCGCATTGACCGCCTTAAGTTTTTCTCTGATTATCTTATTGTTTCAGTATTTAGGATTCGGAAAGACATTTACAGCAGAACAGCTGATCTTCCATCTGGGATTTTTAGGGGTGTGCATGCTGGGCGGGATTTTCGGCGTGAACATGAGGGGAAACAGGTCTTCCACATAAAAAAGAGCGGGCTCACTTGCCCGCTCTTTTTTGTTTGCTATGTATAGCTCCTGCTG |
| chloramphenicol resistance and P*_43_* promoter | GAGCGGATAACAATTTCACACAGGAAACAGCTATGACCATGATTACGAATTCGAGCTCGGTACCCGGGGATCCTCTAGAGATTGTACCGTTCGTATAGCATACATTATACGAAGTTATGCCATAGTGACTGGCGATGCTGTCGGAATGGACGACGGCAATAGTTACCCTTATTATCAAGATAAGAAAGAAAAGGATTTTTCGCTACGCTCAAATCCTTTAAAAAAACACAAAAGACCACATTTTTTAATGTGGTCTTTTATTCTTCAACTAAAGCACCCATTAGTTCAACAAACGAAAATTGGATAAAGTGGGATATTTTTAAAATATATATTTATGTTACAGTAATATTGACTTTTAAAAAAGGATTGATTCTAATGAAGAAAGCAGACAAGTAAGCCTCCTAAATTCACTTTAGATAAAAATTTAGGAGGCATATCAAATGAACTTTAATAAAATTGATTTAGACAATTGGAAGAGAAAAGAGATATTTAATCATTATTTGAACCAACAAACGACTTTTAGTATAACCACAGAAATTGATATTAGTGTTTTATACCGAAACATAAAACAAGAAGGATATAAATTTTACCCTGCATTTATTTTCTTAGTGACAAGGGTGATAAACTCAAATACAGCTTTTAGAACTGGTTACAATAGCGACGGAGAGTTAGGTTATTGGGATAAGTTAGAGCCACTTTATACAATTTTTGATGGTGTATCTAAAACATTCTCTGGTATTTGGACTCCTGTAAAGAATGACTTCAAAGAGTTTTATGATTTATACCTTTCTGATGTAGAGAAATATAATGGTTCGGGGAAATTGTTTCCCAAAACACCTATACCTGAAAATGCTTTTTCTCTTTCTATTATTCCATGGACTTCATTTACTGGGTTTAACTTAAATATCAATAATAATAGTAATTACCTTCTACCCATTATTACAGCAGGAAAATTCATTAATAAAGGTAATTCAATATATTTACCGCTATCTTTACAGGTACATCATTCTGTTTGTGATGGTTATCATGCAGGATTGTTTATGAACTCTATTCAGGAATTGTCAGATAGGCCTAATGACTGGCTTTTATAATATGAGATAATGCCGACTGTACTTTTTACAGTCGGTTTTCTAACGATACATTAATAGGTACGAAAAAGCAACTTTTTTTGCGCTTAAAACCAGTCATACCAATAAATAACTTCGTATAGCATACATTATACGAACGGTATGATAGGTGGTATGTTTTCGCTTGAACTTTTAAATACAGCCATTGAACATACGGTTGATTTAATAACTGACAAACATCACCCTCTTGCTAAAGCGGCCAAGGACGCCGCCGCCGGGGCTGTTTGCGTTCTTGCCGTGATTTCGTGTACCATTGGTTTACTTATTTTTTTGCCAAGGCTGTAATGGCTGAAAATTCTTACATTTATTTTACATTTTTAGAAATGGGCGTGAAAAAAAGCGCGCGATTATGTAAAATATAAAGTGATAGCGGTACCATTATAGGTAAGAGAGGAATGTACAC |
| *glmM* gene | ATGGGCAAGTATTTTGGAACAGACGGTGTAAGAGGTGTCGCCAATAGTGAGCTTACACCTGAGCTGGCCTTTAAAGTCGGACGTTTCGGCGGTTATGTGCTGACAAAAGACAAACAACGTCCAAAAGTGCTGATAGGCCGCGATACACGCATCTCCGGCCATATGCTGGAGGGAGCCCTTGTCGCCGGACTTTTATCCATTGGCGCAGAAGTCATGCGCCTGGGTGTCATTTCTACACCAGGTGTATCTTATTTGACAAAAGCGATGGATGCAGAGGCGGGCGTCATGATTTCCGCTTCTCATAACCCAGTGCAGGATAACGGCATCAAATTCTTTGGGGGAGATGGATTTAAGCTTTCTGATGAACAGGAGGCTGAAATTGAGCGCCTGATGGACGAACCTGAGGATAAGCTGCCAAGACCTGTCGGAGCAGACCTTGGACTTGTAAACGATTATTTTGAAGGCGGACAAAAATATCTGCAATTCTTAAAACAGACAGCTGATGAAGATTTCACAGGCATTCATGTGGCATTGGACTGTGCCAATGGCGCAACGTCATCCTTGGCGACACACCTGTTTGCTGATTTAGATGCAGATGTTTCTACAATGGGGACTTCCCCGAACGGATTAAACATTAATGACGGCGTCGGTTCGACTCATCCCGAAGCGCTCAGCGCGTTTGTCAAAGAGAAAAACGCGGATCTCGGTCTTGCGTTCGACGGTGACGGCGACCGCCTGATTGCTGTCGATGAAAAAGGAAATATTGTAGACGGCGACCAAATCATGTACATATGCTCAAAACATCTGAAATCAGAGGGCCGTTTAAAGGATGATACAGTGGTTTCAACCGTGATGAGCAACCTCGGCTTCTATAAGGCGCTCGAAAAAGAAGGCATCAAAAGCGTGCAGACAGCTGTCGGCGACCGCTACGTAGTAGAAGCAATGAAAAAAGACGGCTACAACGTCGGCGGAGAGCAGTCAGGACATCTTATTTTCCTTGATTACAACACGACAGGGGACGGATTATTGTCTGCTATTATGCTGATGAACACTTTAAAAGCAACAGGCAAGCCGCTGTCAGAGCTTGCAGCTGAAATGCAGAAGTTCCCGCAGCTGTTAGTCAATGTGAGAGTGACTGATAAATATAAAGTTGAAGAAAATGAAAAAGTAAAAGCAGTTATTTCTGAAGTTGAAAAAGAAATGAACGGCGACGGCCGGATTTTGGTGCGCCCTTCAGGAACTGAACCGCTCGTCCGTGTCATGGCTGAAGCGAAGACGAAAGAGCTGTGCGATGAGTATGTCAATCGCATTGTTGAAGTCGTCCGGTCAGAAATGGGATTAGAGTAA |
| right homology arm | TTATTCAGCAGCAGAAACTTCTCTGATCGCGCGGCGGTCGAACGTAAGACGTGTGTTATCACCAGTCTTAATTACGACTTTGCTTTCGTCGATAGAGTCAACAGTACCGTGCAATCCCCCGATTGTCACTACTGAATCGCCTTTTTTCAGTTCCTCTTGCATTTGGCGTACAGCCTTTTGCTGCTTTTGCTGAGGACGAATCAGCAAGAAGTAAAGGACCGCAAACATTAAAATAATAGGAACCAATGTACCTAAAGTGCCAGTCATCATTTTCACCCCTTTCTGTAAGCCTGTTATGTTTATTTAGAAGCTTTTCGCATTTGGCTTGTTATAGCCGTAACGCTCAAAGAACTCTTCTCTGAAATCACCCAGACGATCCTCGCGAATAGCCTGTCTTACCTGCTCCATTAAGTGTAACAGAAAATGAAGGTTATGGTAAGTTGTTAATCGAAGGCCGAATGTTTCATTGCAGCGAATCAAGTGCCTAATGTACGCGCGAGTGTAGTTTTTACATGTATAGCAATCACACTCTTCATCAATCGGACGGAAGTCTCTTTCAAATTTCGCATTTTTCATATTGAGTCGTCCTTCAGCAGTAAACACTGTGCCGTTTCTTGCAATCCGTGTAGGCAGCACACAGTCAAACATATCGACCCCGCGGATCGCTCCGTCGATCAGTGCGTCCGGAGAACCTACTCCCATCAGATATCTCGGTTTATCCTTCGGCAAGAGCGGTGTTGTGAATTCGAGTACACGGTTCATGACATCCTTCGGTTCACCTACAGACAATCCGCCTATAGCATATCCCGGAAAATCAAGCGAAATCAAGTCCTTCGCACTTTGTGTCCGCAGATCTTCATATTCTCCGCCTTGTACAATACCAAACAGCCCTTGTTCATCCTGACGATTGTGGGCATTCAGGCAGCGTTCCGCCCAGCGGCTTGTCCGTTCAACTGACCTCTTCATGTAGTCGTACTCCGCCGGGTATGGCGGGCATTCGTCAAACGCCATCATAATATCAGAGCCGAGTGCGTTTTGGATC |

**Table S14 Genomic locus of *glmU* gene integration expression**

| **Name** | **Content (5’→3’)** |
| --- | --- |
| left homology arm | GCGAACAGGCATCCTATACACTGGGACAATTGATATTCGCTGTGGCGCTGTTTACTGTGGCGCTCTTTAACTACAATCTGATCGCGGGCTTTATTTGGGACCGGATGAAGAAGGTGCTGCGTCACGAATATTTCGTCCACAGCACCTCGCATATTACACATGCAACCATCATGGCGATCATCATTGTGCCGCTGTTCTTCTTGATATTTACAGTGGTCTATCATAAGAGAACGAAACCGATCGGAGAGAAAGCTGACCCTGAGCGTCTTGCTGCGTTTCTCAATGAAAAAGGCGGCAACGCGCTGAGCCATCTTGGTTTTCTTGGAGATAAGCGGTTTTATTTTTCTAGCGATGGAAATGCACTGCTTCTGTTTGGGAAAATCGCCAGAAGGCTGGTCGTGCTCGGCGATCCATCTGGCCAAAGAGAATCATTCCCGCTCGTGCTGGAAGAATTTCTGAACGAAGCGCATCAGAAGGGATTCAGTGTTTTGTTCTATCAAATTGAACGAGAGGACATGGCGCTGTATCACGATTTTGGCTACAACTTCTTTAAATTGGGTGAGGAAGCATATGTAGATTTAAATACATTTACCTTGACTGGGAAGAAAAAAGCCGGCCTTCGGGCAATCAATAACCGCTTTGAGCGGGAGGAGTATACTTTCCATGTGGATCATCCCCCATTTTCTGATGCGTTTTTGGAGGAGCTGAAGCAAATCTCAGACGAATGGCTCGGCTCGAAAAAAGAGAAGGGATTCTCGCTCGGATTTTTTGATCCTTCCTATTTACAGAAAGCGCCGATCGCCTATATGAAAAATGCAGAAGGAGAGATCGTTGCATTCGCAAATGTCATGCCGATGTACCAGGAAGGAGAGATATCGGTCGATCTGATGCGCTATCGCGGCGACGCTCCAAATGGCATTATGGACGCATTGTTTATCCGTATGTTTTTATGGGCAAAGGAAGAGGGCTGTACGTCATTTAATATGGGGATGGCACCCTTGGCCAATGTCGGCACTGCCTTTACATCCTTCTGGTCCGAAAGGTTTGCCGCTGTCATTTTTAATAATGTCAGATACATGTACAGTTTCAGCGGCCTAAGAGCCTTTAAAGAAAAATATAAACCGGAGTGGCGAGGGAAATACTTAGCGTATCGGAAAAACAGATCTCTTTCTGTCACCATGTTCCTCGTTACACGTCTGATTGGCAAAAGCAAAAAAGACTCCGTCTAATAA |
| spectinomycin resistance and P*_43_* promoter | GAGCGGATAACAATTTCACACAGGAAACAGCTATGACCATGATTACGAATTCGAGCTCGGTACCCGGGGATCCTCTAGAGATTGTACCGTTCGTATAGCATACATTATACGAAGTTATCGATTTTCGTTCGTGAATACATGTTATAATAACTATAACTAATAACGTAACGTGACTGGCAAGAGATATTTTTAAAACAATGAATAGGTTTACACTTACTTTAGTTTTATGGAAATGAAAGATCATATCATATATAATCTAGAATAAAATTAACTAAAATAATTATTATCTAGATAAAAAATTTAGAAGCCAATGAAATCTATAAATAAACTAAATTAAGTTTATTTAATTAACAACTATGGATATAAAATAGGTACTAATCAAAATAGTGAGGAGGATATATTTGAATACATACGAACAAGTTAATAAAGTGAAAAAAATACTTCGGAAACATTTAAAAAATAACCTTATTGGTACTTACATGTTTGGATCAGGAGTTGAGAGTGGACTAAAACCAAATAGTGATCTTGACTTTTTAGTCGTCGTATCTGAACCATTGACAGATCAAAGTAAAGAAATACTTATACAAAAAATTAGACCTATTTCAAAAAAAATAGGAGATAAAAGCAACTTACGATATATTGAATTAACAATTATTATTCAGCAAGAAATGGTACCGTGGAATCATCCTCCCAAACAAGAATTTATTTATGGAGAATGGTTACAAGAGCTTTATGAACAAGGATACATTCCTCAGAAGGAATTAAATTCAGATTTAACCATAATGCTTTACCAAGCAAAACGAAAAAATAAAAGAATATACGGAAATTATGACTTAGAGGAATTACTACCTGATATTCCATTTTCTGATGTGAGAAGAGCCATTATGGATTCGTCAGAGGAATTAATAGATAATTATCAGGATGATGAAACCAACTCTATATTAACTTTATGCCGTATGATTTTAACTATGGACACGGGTAAAATCATACCAAAAGATATTGCGGGAAATGCAGTGGCTGAATCTTCTCCATTAGAACATAGGGAGAGAATTTTGTTAGCAGTTCGTAGTTATCTTGGAGAGAATATTGAATGGACTAATGAAAATGTAAATTTAACTATAAACTATTTAAATAACAGATTAAAAAAATTATAAATAACTTCGTATAGCATACATTATACGAACGGTAGAATCGTCGACTGATAGGTGGTATGTTTTCGCTTGAACTTTTAAATACAGCCATTGAACATACGGTTGATTTAATAACTGACAAACATCACCCTCTTGCTAAAGCGGCCAAGGACGCCGCCGCCGGGGCTGTTTGCGTTCTTGCCGTGATTTCGTGTACCATTGGTTTACTTATTTTTTTGCCAAGGCTGTAATGGCTGAAAATTCTTACATTTATTTTACATTTTTAGAAATGGGCGTGAAAAAAAGCGCGCGATTATGTAAAATATAAAGTGATAGCGGTACCATTATAGGTAAGAGAGGAATGTACAC |
| *glmU* gene | ATGGATAAGCGGTTTGCAGTTGTTTTAGCGGCTGGACAAGGAACGAGAATGAAATCGAAGCTTTATAAAGTCCTTCATCCAGTTTGCGGTAAGCCTATGGTAGAGCACGTCGTGGACGAAGCCTTAAAATTATCTTTATCAAAGCTTGTCACGATTGTCGGACATGGTGCGGAAGAAGTGAAAAAGCAGCTTGGTGATAAAAGCGAGTACGCGCTTCAAGCAAAACAGCTTGGCACTGCTCATGCTGTAAAACAGGCACAGCCATTTCTTGCTGACGAAAAAGGCGTCACAATTGTCATTTGCGGAGATACGCCGCTTTTGACAGCAGAGACGATGGAACAGATGCTGAAAGAACATACACAAAGAGAAGCGAAAGCTACGATTTTAACTGCGGTTGCAGAAGATCCAACTGGATACGGCCGCATTATTCGCAGCGAAAACGGAGCGGTTCAAAAAATAGTTGAGCATAAGGACGCCTCTGAAGAAGAACGTCTTGTAACTGAGATCAACACCGGTACGTATTGTTTTGACAATGAAGCGCTATTTCGGGCTATTGATCAGGTGTCTAATGATAATGCACAAGGCGAGTATTATTTGCCGGATGTCATAGAGATTCTTAAAAATGAAGGCGAAACTGTTGCCGCTTACCAGACTGGTAATTTCCAAGAAACGCTCGGAGTTAATGATAGAGTTGCTCTTTCTCAGGCAGAACAATTTATGAAAGAGCGCATTAATAAACGGCATATGCAAAATGGCGTGACGTTGATTGACCCGATGAATACGTATATTTCTCCTGACGCTGTTATCGGAAGCGATACTGTGATTTACCCTGGAACTGTGATTAAAGGTGAGGTGCAAATCGGAGAAGATACGATTATTGGCCCTCATACGGAGATTATGAATAGTGCCATTGGCAGCCGTACGGTTATTAAACAATCGGTAGTCAATCACAGTAAAGTGGGGAATGATGTAAACATAGGACCTTTTGCTCACATCAGACCTGATTCTGTCATCGGGAATGAAGTGAAGATCGGGAATTTTGTAGAAATTAAAAAGACTCAATTCGGAGACCGAAGCAAGGCATCTCATCTAAGCTATGTCGGCGATGCTGAGGTAGGCACTGATGTAAACCTGGGCTGCGGTTCAATTACTGTCAATTATGATGGAAAGAATAAGTATTTGACAAAAATTGAAGATGGCGCGTTTATCGGCTGCAATTCCAACTTGGTTGCCCCTGTCACAGTCGGAGAAGGCGCTTATGTGGCGGCAGGTTCAACTGTTACGGAAGATGTACCTGGAAAAGCACTTGCTATTGCCAGAGCGAGACAAGTAAATAAAGACGATTATGTGAAAAATATTCATAAAAAATAA |
| right homology arm | GACGGAGTCTTTTTTTATTTCGTTTTTAAGAAGTAGGTTTCAATATCATCAAGCAGGTAGTTTGCGGCTTTAATTCCGCCTGCGGTTGTCCATACGACGTCATCGACTTCATGTGCATTACCTGACTTCACGGCCTTCAGGTTTTTCCATAGTGAACTGCTTGTCCACTGATTGGCCCATTTTTCATTTTCTTTTGCATTATCGGCTTTATAAGTGAAATAAAACAGCACATCAGCATCCATATCAGGAATAGATTCTTTACTGTCGGTTGAGAACGTAAATTGGTCCTTTTGTTTCTTAAACAGTTCCACTTGTTTTTCAGGGCGTTTGAAGCCAAGCTGGTCTAAAATAATCCCAGGAAATGAATCGGTATAATAGATTCTTGATTCACCGGATAAAAAGCGTACGACTGAAACGGTTTTGTTTGTCTGGTCGCCCAGCTTGTTTTTTAAATCAGAAACACGTTTATCAAAATCGGCAATGACTTCCTTGCCTTTGTCCGCCTTATTCACCGCGTTGGCGTATAGAGTCAGGTTATCCTTCCAGTTCCCGGCCAAAGATTCAGCAAAAACAGTCGGCGCAATCGCATTTAACTGATCATAAATTTTTTCCTGGCGCACTTTGTTTCCAATAATCAAATCAGGCTTTAATTCTGCGATGGCTTCCACATTCGGTTCCGTTTCAAGACCGACGTTTTTAACACCTTTCATGTCATCTTTCAGATAGTCATACCACGGGTCGCCCTTCCATGACTTTACGGCTCCGACCGGCTTAATGCCAAGCGCTAAGAGCGCCTCTGTTCCTTCATTAGTTAAAACAACAATCCGTTTTGGATTGGCGGGGATGTTGTCTGATGTCCCCATTGCATGTTTGACCGTTCTTGTTTTGCTGCTTGACACCTCGCTGTTGCTTGAACTTTCTGAACTATTACAGGCAGAAAGTACCATAACGGCCATTAAAAATACGAATAGCATGCTGATATGCTTTTTCATCTTTTTCCTCCCAATATTGAAATTCATTATCATTTAGATCATAATAAGCAGTGTTAAGAGTGTCAATCCCTAATTGAGGATTATTCTCAAAAACAAACATTACATAGTAAATAACTAGGAGAATTAGATCATGATCTGCAAAAAGGCATCTTCAAAATGGATTGTGTTAG |
